# Anatomic, computed tomographic, and ultrasonographic assessment of the lymph nodes in presumed healthy adult cats: the abdomen, pelvis, and hindlimb

**DOI:** 10.1186/s13028-022-00638-x

**Published:** 2022-08-13

**Authors:** Mauricio Tobón Restrepo, Rosa Novellas, Adrià Aguilar, Xavier Moll, Yvonne Espada

**Affiliations:** 1 Departament de Medicina I Cirurgia Animals, Facultat de Veterinària, Universitat Autònoma de Barcelona, Travessera dels Turons, 08193 Bellaterra, Spain; 2 Present Address: Department of Clinical Sciences of Companion Animals, Faculty of Veterinary Medicine, Utrecht University, Yalelaan 108, 3584 CM Utrecht, The Netherlands

**Keywords:** Abdominal, Computed tomography, Feline, Lymph nodes, Imaging

## Abstract

**Background:**

The computed tomography (CT) and ultrasonography (US) features of lymph nodes of the abdomen, pelvis, and hindlimb in healthy cats are poorly described in the current literature. A prospective anatomic and reference interval study was therefore performed. The lymph nodes of six feline cadavers were identified, and dimensions were measured (length, width, and height). The lymph nodes from 30 healthy adult cats were identified and measured using CT (pre- and postcontrast) and US. The identification and dimensions of the separate lymph nodes were compared between imaging techniques and the anatomic study.

**Results:**

The identification of lymph nodes was most frequent in CT, and the dimensions were overall larger than those identified and measured in US and the anatomic study. The caudal epigastric and sacral lymph nodes were not identified in the anatomic study. The ischiatic, lumbar aortic, internal iliac, and caudal epigastric lymph nodes were not visualized in US. The height presented the main statistical differences among techniques. The lymph nodes were mainly homogeneous in pre- and postcontrast CT and US images. Some lymph nodes showed a hyperattenuating periphery with a hypoattenuating center (on pre- and postcontrast images) and a hypo-/isoechoic periphery with a hyperechoic center, representing the hilar fat. The lymph nodes were commonly elongated and rounded except for the jejunal lymph nodes, which had an irregular shape.

**Conclusions:**

The assessment of most of the abdominal, pelvic, and hindlimb lymph nodes in the cat is feasible using CT and US, with CT performing best. Factors like the amount of adipose tissue and contrast administration subjectively improved the lymph node visualization and assessment. The measurements and features reported are proposed as reference values.

**Supplementary Information:**

The online version contains supplementary material available at 10.1186/s13028-022-00638-x.

## Background

The abdominal lymph centers in the cat are divided into parietal and visceral groups as in dogs [1, 2]. The parietal group has four lymph centers (lumbar, iliosacral, inguinofemoral, and ischiatic), and the visceral group has three lymph centers (celiac, cranial and caudal mesenteric) [3, 4]. Previous studies regarding the ultrasonographic assessment of the abdominal cavity in the cat reported that the most frequently identified lymph nodes from the visceral group are the gastric, hepatic, pancreaticoduodenal, jejunal, ileocecal, and colic lymph nodes; and from the parietal group are the medial iliac and the superficial inguinal lymph nodes [5]. The lymph nodes less frequently identified in the visceral group are the splenic and caudal mesenteric lymph nodes, and in the parietal group are the lumbar aortic, renal, internal iliac (formally called hypogastric [4]), sacral, and caudal epigastric lymph nodes [5, 6]. The peripheral lymph nodes of the abdomen are described as fusiform and slender in shape with ultrasonography (US). When compared with surrounding fat tissue, they appeared slightly rounded, with regular margins and hypoechoic. Deep abdominal lymph nodes were described as more rounded and elongated in shape, slightly hypoechoic to the surrounding peritoneum, and with regular margins [5, 7]. The iliofemoral and the popliteal lymph centers have been identified as the two lymph centers for the cat’s hindlimb [3]. In ultrasound, the popliteal lymph node is described as an oval or rounded node of variable size (short axis range of 2.8–6.5 mm; long axis range of 4.3–12.0 mm) [8].

There is scarce literature about the normal computed tomographic appearance of the abdominal lymph nodes in the cat. In dogs, abdominal lymph nodes have been described as homogeneously attenuating structures commonly elongated in shape. Some lymph nodes were slightly irregular or relatively more hyperattenuating in the periphery than centrally before and after contrast administration due to the presence of fatty tissue in the hilus [2].

To the authors’ knowledge, studies comparing the identification, dimensions and features of abdominal and hindlimb lymph nodes between gross dissection, CT, and US are lacking.

The aims of this study were: (i) to describe the features of the abdominal, pelvic, and hindlimb lymph nodes in CT and US in a group of healthy adult cats, and (ii) to compare the identification and dimensions of the lymph nodes from the abdomen and hindlimb obtained with US and CT in a group of healthy adult cats with those obtained from an anatomic study.

## Methods

### Anatomical study

Cadavers of cats referred to the pathology department of the Universitat Autònoma de Barcelona from January 2013 to June 2015 were prospectively included. Exclusion criteria were: time of death > 24 h, age < 1-year, and a cause of death due to inflammatory or neoplastic conditions. The cadavers were included if owner consent was obtained.

The dissections were performed by one author (MTR). A small incision was made immediately caudal to the xiphoid process in the *linea alba* with a 24-scalpel blade. The incision was continued caudally with Mayo scissors until the pubic bone. Afterward, the abdominal muscles were cut following the costal arch until the spine. Then, blunt dissection of the abdominal lymph nodes was performed with special care in not tearing any great vessels. Then, the skin was removed from the hindlimbs with an incision in the cranial aspect of the thigh until the talus. After that, the incision surrounded the talus, and the skin was pulled off. The lymph nodes were searched following the previous anatomic descriptions [3, 5, 9]. The lymph nodes were measured using a manual dial caliper (Vernier 0–150 mm/0.02 high precision). The length was defined as the largest dimension in the craniocaudal plane. For the lymph nodes of the cranial mesenteric lymph center, the length was measured following a parallel direction with the gastrointestinal tract or the jejunal vessels. The height was obtained at the thickest point in the dorsoventral plane, and the width was measured at the thickest point in the mediolateral plane. A short-to-long axis ratio was calculated with the length and height to determine the shape. As previously described, a lymph node was classified as rounded when the short-to-long axis ratio was > 0.5, and elongated when the short-to-long axis ratio was ≤ 0.5 [2, 10]. Multilobular lymph nodes that did not fit the ratio were classified as miscellaneous. The number, anatomical landmarks, shape, and size of lymph nodes per lymph center were recorded.

### Imaging study

Clinically healthy cats older than 1 year were prospectively recruited from October 2013 to July 2015. All animals belonged to staff, students, and hospital clients at the Fundació Hospital Clinic Veterinari of the Universitat Autònoma de Barcelona. The owner’s writing consent was obtained for all the included cats. Clinically healthy status was determined based on physical examination (performed by AA and XM), biochemical profile [glucose, total proteins, cholesterol, calcium, potassium, alanine-amino-transferase (ALT), gamma-glutamyl-transferase (GGT), creatinine, urea], complete blood count, fast test to rule out feline immunodeficiency virus antibodies and feline leukemia virus antigens, and a polymerase chain reaction test for *Bartonella* sp. as described previously by the authors [11]. To perform CT and US, the patients underwent sedation using midazolam (0.2 mg/kg) (Midazolam 15 mg/3 mL, Normon, Spain), butorphanol (0.4 mg/kg) (Torbugesic 10 mg/mL, Zoetis, Madrid, Spain), and ketamine (5 mg/kg) (Imalgene 100 mg/mL, Merial, Barcelona, Spain). Anesthesia was induced and maintained with Isoflurane (1.5–5% dosage 100% O_2_ at 4 L/min) (Isoflurane, Abbott Laboratories, Berkshire, UK). CT and US images were acquired and reviewed by a Ph.D. candidate/veterinarian (MTR) under the supervision of a board-certified veterinary radiologist (RN) and a radiology professor (YE).

### Computed tomography

A whole-body CT scan was performed with the cats positioned in dorsal recumbency with the forelimbs and hindlimbs along the sides. Dorsal recumbence was performed for optimal image comparison between CT and US and the anatomical study in which the cadavers were in a similar position. Scans were performed in a 16-slices helical CT-scanner (GE^®^ Brivo CT 385, Madrid, Spain). Images are acquired in a soft tissue algorithm before and after intravenous administration of 600 mg/kg of Iopromide (300 mgI/mL) (Ultravist^®^ 300 mg/mL, Bayer Pharma AG, Berlin, Germany) or Iopamidol (300 mgI/mL) (Scanlux^®^ 300 mg/mL, Sanochemia pharmazeutika, Neufeld/Leitha, Austria) via the cephalic vein. Technical details were: slice thickness of 0.625 mm, interval thickness of 0.625 mm, collimation pitch of 1.25 mm, 120 kV, 50–90 mA, and matrix of 512 × 512. In this study, the assessment of the abdominal cavity to identify the lymph nodes was performed following anatomic references and available literature [3, 4, 11, 16]. Using multiplanar reconstruction on CT images, the lymph nodes along the intestinal tract (e.g., colic lymph node) or along the great vessels of the abdomen (e.g., medial iliac lymph node) were numbered from oral to aboral and from cranial to caudal, respectively. The jejunal lymph node at the medial or dorsal aspect of the jejunal vessels was numbered as one and at the lateral or ventral as two. The most ventral ileocecal lymph node was numbered as one and the dorsal as two. All these indications were also used during the US examination and in the anatomic study to accurately compare the different lymph nodes.

The CT dimensions and features of each identified lymph node were performed following previously described methods for measuring and comparing CT images [2, 9, 11]. All recollected data were recorded using an image archiving and communication system software (Centricity PACS-IW, General Electric^®^ Healthcare). Measurements were performed as previously described for dogs [2, 9]. Two previously reported methods were used to determine the length; (1) calculated length: multiplying the number of transverse images that contained the lymph node by the slice thickness; and (2) multiplanar reconstruction-length (MPR-length): a sagittal image of the lymph node at its maximal dimension was generated with multiplanar reconstruction, an electronic caliper was placed from the cranial to the caudal border to measure the length of the lymph node. Width and height were measured in transverse images at the cranial, middle, and caudal aspects of the lymph nodes. The width was defined as the distance from the medial to the lateral border, and height was defined as the distance from the ventral to the dorsal border in each position. The highest values were used for the statistical analysis. The short-to-long axis ratio was calculated in each lymph node using the higher value of height divided by the value of length obtained in the multiplanar reconstruction. The shape was then classified according to the ratio in the same way as explained in the anatomic study. A 2–4 mm^2^ circular/oval region of interest (ROI) was placed over the same cranial, middle, and caudal transverse slices (where width and height measurements were performed) to calculate attenuation (Hounsfield units) values. Attenuation measurements were performed in both pre- and postcontrast images. Mean values for attenuation pre- and postcontrast were calculated using the three previously obtained measures. As in previously reported studies, lymph nodes attenuation was compared with the surrounding muscles and was classified as isoattenuating, slightly hypoattenuating (slightly less attenuating and homogeneous), hypoattenuating (markedly less attenuating and homogeneous), hyperattenuating and heterogeneous (single or multiple areas of different attenuation within the lymph node). On postcontrast images, the attenuation was classified as homogeneous, mildly heterogeneous (small, multiple areas of varying contrast enhancement), heterogeneous (large, multiple areas of varying contrast enhancement), and peripheral enhancement (contrast enhancement in a ring-like distribution with a hypoattenuating center).

### Ultrasonography

Ultrasonographic examination was performed in all cats after the CT scan with the maintenance of general anesthesia. The animals were positioned in dorsal recumbency (similar to CT), and the hair of the abdomen and the caudal aspect of each stifle was clipped. B-Mode ultrasonography was performed using an Esaote Mylab70 Xvision^®^ (Firenze, Italy) ultrasound machine with a 4–13 MHz frequency linear transducer. Settings on the machine were adjusted to optimize image acquisition for each patient, including depth, focus, gain, and frequency. Acoustic coupling gel (Transonic gel^®^, Telic, Barcelona, Spain) was generously applied to ensure adequate skin-transducer contact.

For the sagittal plane, the transducer was placed with the guide pointing cranial and parallel to the spine. An image including the largest measurement of the lymph node was recorded. For the lymph nodes of the cranial mesenteric lymph center (e.g., jejunal, colic, ileocecal), the image for the sagittal plane was performed following a parallel direction to the longitudinal axis of the intestines or blood vessels. Measurement in this image was performed using an electronic caliper from the cranial to the caudal border (long axis) of the lymph node and was defined as length. In the same image, a second measurement made perpendicularly to the first one at the point of the maximum thickness (ventral to dorsal) was defined as the height (short axis). As for the anatomic study and the CT images, the short-to-long axis ratio was calculated for each lymph node and used to determine the shape. For the transverse image of the lymph node, the transducer was rotated 90° with the guide towards the patient’s right side to select then an image that contained the largest portion of the lymph node. A measurement was performed from medial to lateral in this image and was considered the width.

For each lymph node, echogenicity was recorded as hypoechoic, isoechoic, hyperechoic, or heterogeneous when compared to surrounding fat tissue. The presence of a hyperechoic central line that corresponded with the hilus was also recorded. The shape of each lymph node was evaluated following the same criteria as in CT. Margins were defined as smooth or irregular.

### Statistics

Statistic tests were selected by one of the authors (MTR) and a statistician (OC). Data were digitalized using Microsoft Excel (Microsoft Office Excel, 2010, USA). Statistical analyses were performed using the free available statistics software R (R version 3.2.3, 2015-12-10), Copyright ©, the R foundation for statistical computing). Descriptive statistics (frequency, mean, median, and standard deviation) of lymph nodes identification, attenuation values in pre- and postcontrast images, echogenicity, and measurements were calculated. Wilcoxon Signed Rank Test was used to compare the pair distribution between the calculated length and the MPR-length obtained of the lymph nodes on CT images, as well as MPR-length, height, and width between CT and US. Mann–Whitney U test was used to compare the pair distribution of the lymph nodes measurements (MPR-length, width, and height) between CT and anatomy; and between US and anatomy. Each measurement was compared individually for each lymph center and not for the whole sample of identified lymph nodes (No Post-Hoc corrections were used). A P value < 0.05 was considered statistically significant.

## Results

Anatomic study: six feline cadavers were included. Two cats died of heart failure, two of kidney failure, one due to poisoning, and one of unknown cause. The average age was 6.8 years (range 1–16). The breed includes five domestic short-hair and one British long-hair cat.

Imaging study: Thirty-four cats were recruited, but four were excluded due to a positive test result for *Bartonella* sp. (3/4) and feline immunodeficiency virus antibodies (1/4), resulting in a final study sample of 30 presumed clinically healthy cats. The average age was 3.7 years (range 1.5–17), and the average weight was 4.4 kg (range 3.0–7.0). Twenty-nine cats were domestic short-hair, and one cat was a Persian. The study population consisted of 16.7% males (n = 5), 20.0% neutered males (n = 6), 30.0% females (n = 9), and 33.3% neutered females (n = 10).

Table 1 shows the frequency of identification of lymph nodes per technique. Tables 2, 3 and 4 summarizes the mean and standard deviation for the length, width, and height together with the results of the statistical comparisons among techniques. Additional file 1 shows the attenuation values (Hounsfield units) of the lymph nodes. Additional file 2 summarizes the echogenicity of the lymph nodes, and Additional file 3 summarizes the shape of the lymph nodes per technique.

**Table 1:** Frequency of lymph node identification in each technique

Lymph center	Lymph node	Frequency						Lymph center	Lymph node	Frequency					
		CT		US		Anatomy				CT		US		Anatomy	
		n	%	n	%	n	%			n	%	n	%	n	%
Celiac	Gastric 1	28	93.33	27	90.00	5	83.33	Lumbar	Right renal	7	23.33	2	6.67	2	3.33
	Gastric 2	6	20.00	2	6.67	0	0.00		Left renal	4	13.33	0	0.00	2	33.33
	Hepatic	22	73.33	20	66.67	5	83.33	Iliosacral	Right medial iliac	28	93.33	30	100.00	5	83.33
	Splenic	23	76.67	23	76.67	2	33.33		Left medial iliac	28	93.33	30	100.00	5	83.33
	Pancreaticoduodenal	29	96.67	29	96.67	6	100.00		Right internal iliac	19	63.33	0	0.00	1	16.67
Cranial mesenteric	Jejunal 1	30	100.00	30	100.00	6	100.00		Left internal iliac	19	63.33	0	0.00	0	0.00
	Jejunal 2	28	93.33	28	93.33	5	83.33		Right sacral	8	26.67	1	3.33	0	0.00
	Jejunal 3	14	46.67	15	50.00	4	66.67		Left sacral	4	13.33	0	0.00	0	0.00
	Jejunal 4	0	0.00	0	0.00	3	50.00	Inguinofemoral	Right Superficial inguinal	28	93.33	28	93.33	4	66.67
	Ileocecal 1	27	90.00	30	100.00	6	100.00		Left superficial inguinal 1	28	93.33	29	96.67	4	66.67
	Ileocecal 2	27	90.00	24	80.00	4	66.67		Left superficial inguinal 2	1	3.33	0	0.00	0	0.00
	Colic 1	27	90.00	21	70.00	6	100.00		Right superficial caudal epigastric 1	26	86.67	0	0.00	0	0.00
	Colic 2	15	50.00	0	0.00	6	100.00		Right superficial caudal epigastric 2	4	13.33	0	0.00	0	0.00
	Colic 3	9	30.00	0	0.00	4	66.67		Right superficial caudal epigastric 3	1	3.33	0	0.00	0	0.00
	Colic 4	5	16.67	0	0.00	4	66.67		Left superficial caudal epigastric 1	26	86.67	0	0.00	0	0.00
	Colic 5	4	13.33	0	0.00	2	33.33		Left superficial caudal epigastric 2	2	6.67	0	0.00	0	0.00
Caudal mesenteric	Caudal mesenteric 1	29	96.67	10	33.33	5	83.33	Ischiatic	Right ischiatic	6	20.00	0	0.00	1	16.67
	Caudal mesenteric 2	14	46.67	0	0.00	4	66.67		Left ischiatic	3	10.00	0	0.00	1	16.67
	Caudal mesenteric 3	5	16.67	0	0.00	2	33.33	Popliteal	Right popliteal	29	96.67	30	100.00	6	100.00
	Caudal mesenteric 4	2	6.67	0	0.00	1	16.67		Left popliteal	29	96.67	29	96.67	6	100.00
Lumbar	Lumbar aortic 1	4	13.33	0	0.00	3	50.00								
	Lumbar aortic 2	0	0.00	0	0.00	2	33.33								
	Lumbar aortic 3	0	0.00	0	0.00	1	16.67

**Table 2:** Mean and standard deviation for length, width, and height of the lymph nodes of the celiac and cranial mesenteric lymph centers

Lymph center	Celiac					Cranial mesenteric										
Lymph node	Gastric 1	Gastric 2	Hepatic	Splenic	Pancreatico-duodenal	Jejunal 1	Jejunal 2	Jejunal 3	Jejunal 4	Ileocecal 1	Ileocecal 2	Colic 1	Colic 2	Colic 3	Colic 4	Colic 5
Lengthmm																
CT-Calc	6.34 (2.62)	4.48 (0.74)	9.14 (3.26)	5.08 (1.63)	7.20 (2.18)	34.50 (7.80)	23.60 (7.20)	12.10 (3.40)	–	7.82 (2.52)	7.44 (2.76)	12.58 (4.00)	8.63 (2.49)	6.88 (3.25)	4.76 (1.16)	5.80 (2.42)
CT-MPR	6.58 (3.18)	6.12 (3.91)	9.03 (2.77)	5.33 (2.01)	6.38 (1.82)	36.97 (5.42)	26.73 (4.75)	13.80 (2.50)	–	7.38 (2.21)	7.1 (1.96)	11.86 (3.71)	7.57 (2.51)	5.98 (1.92)	4.06 (0.77)	5.20 (3.52)
US	5.99 (2.05)	4.00 (0.28)	6.15 (2.32)	5.41 (2.03)	5.76 (1.49)	25.05(6.19)	17.87 (7.74)	10.00 (2.34)	–	7.13 (2.65)	6.55 (1.92)	8.14 (2.39)	–	–	–	–
Anatomy	8.56 (6.45)	–	10.84 (2.17)	4.35 (2.62)	5.60 (3.06)	45.30 (10.83)	26.78 (12.45)	11.08 (1.50)	18.67 (18.58)	5.78 (2.37)	5.10 (0.88)	15.68 (10.59)	8.75 (3.62)	6.85 (1.45)	6.70 (3.07)	4.85 (2.89)
MPR vs. Calc					A**	A*	A*						A*			
CT-MPR vs US			A*			A*	A*	A*				A*				
CT-MPR vs Anat										B*						
US vs Anat			B**								B*					
Width mm																
CT	3.30 (1.06)	3.23 (1.11)	3.89 (1.67)	4.12 (1.32)	6.05 (1.79)	9.55 (4.44)	9.58 (4.91)	10.25 (3.06)	–	5.37 (1.54)	7.57 (9.06)	5.94 (3.02)	5.09 (1.91)	4.58 (1.50)	5.40 (1.79)	4.20 (1.68)
US	4.87 (1.16)	3.50 (2.40)	5.33 (1.95)	3.80 (1.31)	5.77 (2.13)	10.93 (5.23)	15.27 (6.04)	8.68 (4.69)	–	6.36 (2.84)	5.96 (2.32)	7.44 (3.38)	–	–	–	–
Anatomy	3.14 (1.05)	–	4.20 (2.02)	2.65 (1.20)	2.55 (1.05)	7.90 (3.50)	5.40 (1.31)	3.75 (0.71)	3.67 (1.80)	2.95 (0.91)	3.45 (1.05)	3.05 (0.63)	4.08 (1.57)	3.48 (1.12)	3.40 (0.89)	3.55 (1.77)
CT vs US	A**						A*									
CT vs Anat					B**		B*	B*		B*	B**	B*				
US vs Anat	B*				B**		B*	B*		B*	B**	B*				
Heigth mm																
CT	3.04 (1.27)	3.43 (0.86)	4.48 (1.62)	3.55 (1.38)	4.31 (1.36)	6.27 (2.58)	5.78 (1.70)	8.53 (4.11)	–	3.80 (1.20)	6.41 (9.8)	5.35 (2.98)	3.70 (1.53)	2.87 (0.92)	3.36 (1.47)	3.30 (1.00)
US	2.50 (0.72)	3.10 (0.28)	2.92 (0.77)	2.53 (0.94)	3.17 (0.94)	4.42 (1.55)	4.23 (1.07)	4.38 (1.75)	–	3.06 (1.01)	2.94 (0.59)	3.68 (1.29)	–	–	–	–
Anatomy	1.1 (0.14)	–	2.44 (1.55)	1.5 (0.71)	1.1 (0.64)	2.50 (1.87)	1.55 (1.05)	1.55 (0.24)	–	1.35 (0.39)	1.47 (0.57)	1.23 (0.46)	1.35 (0.49)	1.30 (0.47)	1.20 (0.18)	1.45 (0.21)
CT vs US	A*			A*	A**		A*	A*		A**	A*					
CT vs Anat	B**		B*	B*	B**	B*	B*	B*		B**	B**	B*	B*	B*	B*	
US vs Anat	B**				B**	B*	B*	B*		B**	B**					

The statistical comparison among techniques is shown

CT: computed tomography, US: ultrasonography, Anat: Anatomy, NC: not calculated, SD: standard deviation, CT-MPR: multiplanar reconstruction-length, CT-Calc.: calculated-length

*P-value < 0.05, **P-value < 0.01, (A) Wilcoxon signed-rank test, (B) Mann–Whitney U test

**Table 3:** Frequency, mean and standard deviation for length, width, and height of the lymph nodes of the caudal mesenteric, lumbar, and iliosacral lymph centers

Lymph center	Caudal mesenteric				Lumbar					Iliosacral					
Lymph node	Caudal mesenteric 1	Caudal mesenteric 2	Caudal mesenteric 3	Caudal mesenteric 4	Lumbar aortic 1	Lumbar aortic 2	Lumbar aortic 3	Right renal	Left renal	Right medial iliac	Left medial iliac	Right internal iliac	Left internal iliac	Right sacral	Left sacral
Length mm															
CT-Calc	11.37 (4.60)	9.44 (4.27)	10.24 (5.01)	12.80 (0.42)	4.25 (1.87)	–	–	9.20 (2.97)	9.25 (2.55)	16.52 (4.78)	17.04 (7.03)	7.96 (2.14)	8.08 (1.88)	5.58 (2.24)	5.65 (1.72)
CT-MPR	10.34 (4.50)	8.98 (4.44)	10.88 (6.26)	13.05 (0.49)	3.93 (1.55)	–	–	9.30 (3.87)	8.80 (2.20)	17.85 (4.85)	17.52 (4.11)	8.17 (2.65)	8.66 (2.60)	5.09 (2.48)	4.65 (1.38)
US	10.00 (2.22)	–	–	–	–	–	–	8.10 (1.70)	–	10.27 (3.56)	11.62 (3.46)	–	–	5.20 (NC)	–
Anatomy	7.34 (4.99)	7.52 (2.23)	6.80 (5.09)	6.70 (–)	3.57 (2.02)	5.40 (3.25)	4.50 (–)	11.40 (8.77)	8.65 (4.60)	12.48 (6.58)	13.28 (4.74)	12.00 (NC)	6.70 (NC)	–	–
MPR vs. Calc															
CT-MPR vs US															
CT-MPR vs Anatomy										B**					
US vs Anatomy															
Width mm															
CT	5.14 (2.21)	5.37 (2.36)	6.38 (2.22)	6.45 (2.05)	2.17 (0.92)	–	–	4.64 (1.61)	3.30 (1.29)	3.46 (1.25)	3.31 (1.00)	4.25 (1.91)	4.07 (1.81)	4.76 (1.83)	4.72 (1.72)
US	7.77 (2.69)	–	–	–	–	–	–	6.10 (2.69)	–	4.64 (1.72)	4.12 (1.33)	–	–	–	–
Anat	3.12 (0.73)	3.12 (0.75)	4.00 (1.70)	4.00 (NC)	1.70 (0.72)	1.95 (0.35)	2.00 (NC)	3.10 (0.42)	2.30 (NC)	3.82 (1.08)	2.88 (0.73)	4.70 (NC)	4.40 (NC)	–	–
CT vs US															
CT vs Anatomy										B**					
US vs Anatomy															
Heigth mm															
CT	3.51 (1.44)	3.53 (1.55)	3.62 (1.82)	4.30 (1.27)	1.82 (1.13)	–	–	3.31 (1.07)	2.95 (1.38)	2.42 (0.65)	2.60 (1.16)	3.10 (1.50)	3.73 (1.71)	2.09 (0.67)	2.17 (0.84)
US	3.50 (0.98)	–	–	–	–	–	–	3.25 (0.64)	–	3.45 (1.34)	3.28 (0.84)	–	–	2.00 (NC)	–
Anatomy	1.66 (0.64)	1.82 (1.20)	1.90 (0.42)	1.90 (NC)	0.87 (0.58)	1.30 (NC)	0.90 (NC)	1.15 (0.21)	1.30 (0.42)	1.40 (0.87)	1.56 (0.88)	2.50 (NC)	–	–	–
CT vs US															
CT vs Anatomy	B*									B*					
US vs Anatomy															

The statistical comparison among techniques is shown

CT: computed tomography, US: ultrasonography, NC: not calculated, SD: standard deviation, CT-MPR: multiplanar reconstruction-length, CT-Calc.: calculated-length

^*^P-value < 0.05, **P-value < 0.01, (A) Wilcoxon signed-rank test, (B) Mann–Whitney U test

**Table 4:** Frequency, mean and standard deviation for length, width, and height of the lymph nodes of the inguinofemoral, ischiatic, and popliteal lymph centers

Lymph center	Inguinofemoral								Ischiatic		Popliteal	
Lymph node	Right superficial inguinal	Left superficial inguinal 1	Left superficial inguinal 2	Right superficial caudal epigastric 1	Right superficial caudal epigastric 2	Right superficial caudal epigastric 3	Left superficial caudal epigastric 1	Left superficial caudal epigastric 2	Right ischiatic	Left ischiatic	Right popliteal	Left popliteal
Length mm												
CT-Calc	9.24 (3.03)	8.81 (4.14)	6.90	12.25 (5.56)	7.83 (3.87)	13.80 (NC)	13.71 (5.35)	15.00 (0.85)	4.92 (1.65)	5.63 (0.65)	8.24 (1.93)	8.14 (2.35)
CT-MPR	9.03 (3.17)	8.12 (3.23)	9.90 (NC)	12.55 (6.98)	7.53 (5.16)	12.70 (NC)	13.37 (5.38)	16.25 (1.63)	4.58 (1.28)	4.63 (0.50)	8.02 (1.78)	8.49 (2.30)
US	7.25 (1.75)	7.21 (2.28)	–	–	–	–	–	–	–	–	7.73 (2.36)	7.88 (2.35)
Anatomy	13.60 (5.62)	13.05 (5.33)	–	–	–	–	–	–	3.20 (–)	3.30 (–)	7.37 (2.28)	6.63 (1.82)
MPR vs. Calc												
MPR-CT vs US	9.24 (3.03)	8.81 (4.14)	6.90	12.25 (5.56)	7.83 (3.87)	13.80 (NC)	13.71 (5.35)	15.00 (0.85)	4.92 (1.65)	5.63 (0.65)	8.24 (1.93)	8.14 (2.35)
MPR-CT vs Anatomy												
US vs Anatomy												
Width mm												
CT	6.88 (2.53)	6.76 (2.95)	6.90 (NC)	5.80 (1.96)	4.30 (0.82)	6.90 (NC)	5.72 (1.95)	5.60 (2.4)	4.05 (0.76)	3.73 (0.91)	6.29 (1.65)	5.97 (1.25)
US	6.19 (3.11)	5.43 (1.82)	–	–	–	–	–	–	–	–	5.99 (1.26)	6.05 (1.32)
Anatomy	3.45 (1.69)	3.27 (1.49)	–	–	–	–	–	–	2.00 (NC)	6.00 (NC)	4.45 (2.52)	4.10 (1.80)
CT vs US												
CT vs Anatomy		B*										
US vs Anattomy		B*										
Heigth mm												
CT	3.21 (1.14)	3.04 (1.28)	4.20 (NC)	3.88 (1.25)	3.83 (1.95)	5.90 (NC)	3.83 (1.35)	5.00 (0.85)	2.42 (0.63)	2.50 (0.1)	5.11 (1.83)	5.42 (1.45)
US	2.72 (0.90)	2.67 (0.62)	–	–	–	–	–	–	–	–	4.99 (1.24)	5.11 (1.36)
Anatomy	1.27 (0.72)	1.27 (0.70)	–	–	–	–	–	–	1.00 (NC)	1.00 (NC)	1.58 (0.93)	1.67 (1.04)
CT vs US												
CT vs Anatomy		B**										
US vs Anatomy		B**										

The statistical comparison among techniques is shown

CT: computed tomography, US: ultrasonography, NC: not calculated, SD: standard deviation, CT-MPR: multiplanar reconstruction-length, CT-Calc.: calculated-length

^*^P-value < 0.05, **P-value < 0.01, (A) Wilcoxon signed-rank test, (B) Mann–Whitney U test

### Lymph center description

#### Celiac lymph center (*Lymphocentrum celiacum*)

**Fig. 1:**
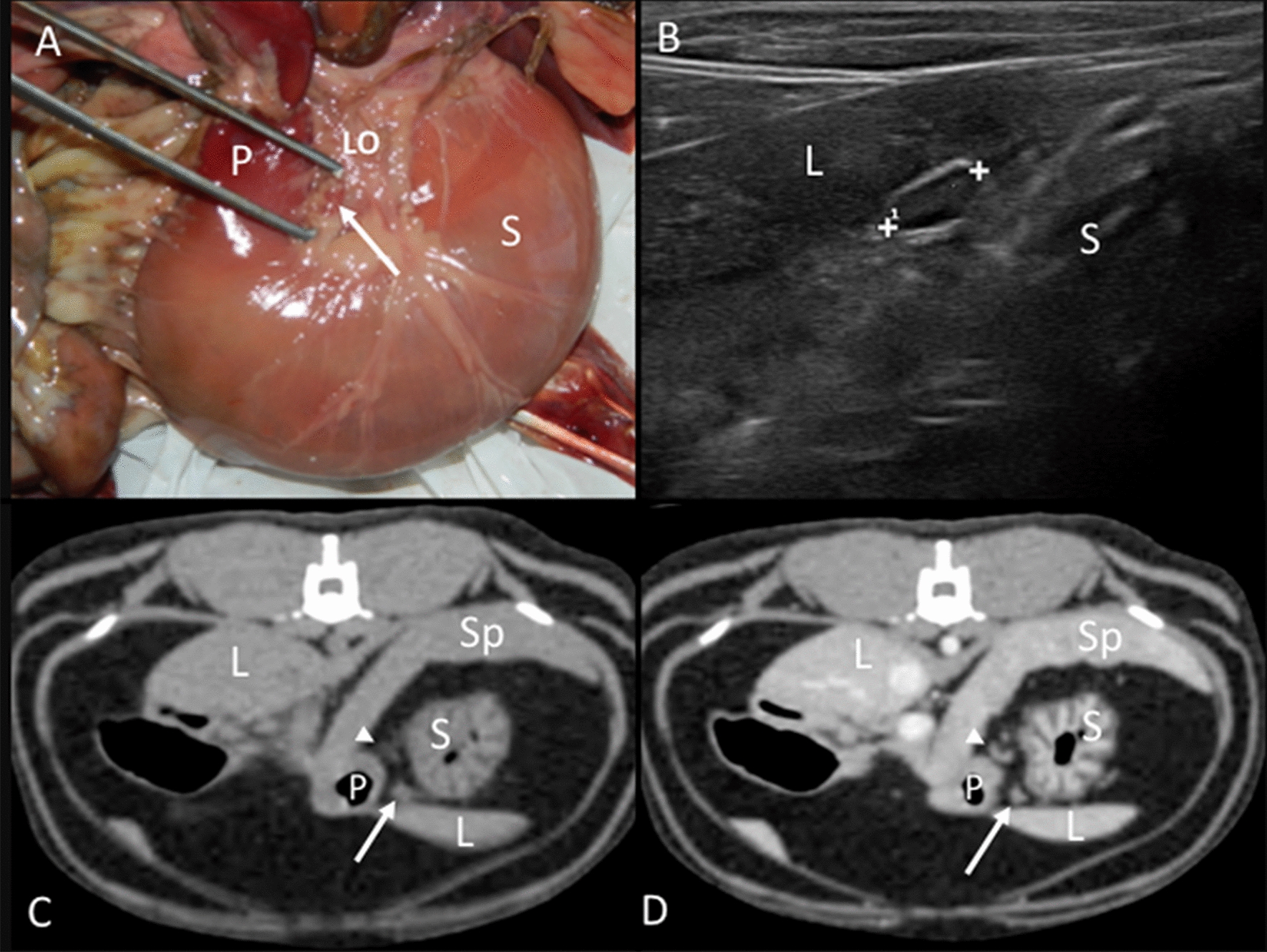
Gastric lymph node. **A** Image of the dissection showing its localization (arrow) in the lesser omentum (LO) of the stomach (S), near the pylorus (P). **B** Ultrasonographic image showing an elongated gastric lymph node (between cursors) with an isoechoic center and a hypoechoic periphery, located between the stomach (S) and the liver (L). **C**, **D** CT images indicating the localization of an isoattenuating gastric lymph node (arrow) in the lesser omentum in the precontrast image (**C**) and with a homogeneous contrast enhancement pattern in the postcontrast image (**D**). A second lymph node (arrowhead) is partially visible dorsally, between the stomach (S) and the spleen (Sp). The liver (L) and pylorus (P) are indicated

Gastric lymph nodes: In the anatomic study, one round or ovoid lymph node was found in the omentum of the gastric lesser curvature (Fig. 1A). A fair amount of fat tissue covered the lymph node in most cases. On CT images, one or two lymph nodes were observed in close contact with the left gastric vein at the gastric lesser curvature (Fig. 1C, D). On the US images, the transducer was placed parallel to the spine immediately caudal to the xiphoid process and then moved slightly to the left until an image with the liver to the left of the screen and the stomach to the right was obtained (Fig. 1B). One or two lymph nodes were in the omentum of the lesser gastric curvature, in the fat tissue between the liver and the stomach. A hyperechoic central line was visible in 33.33% of the identified gastric lymph nodes.

**Fig. 2:**
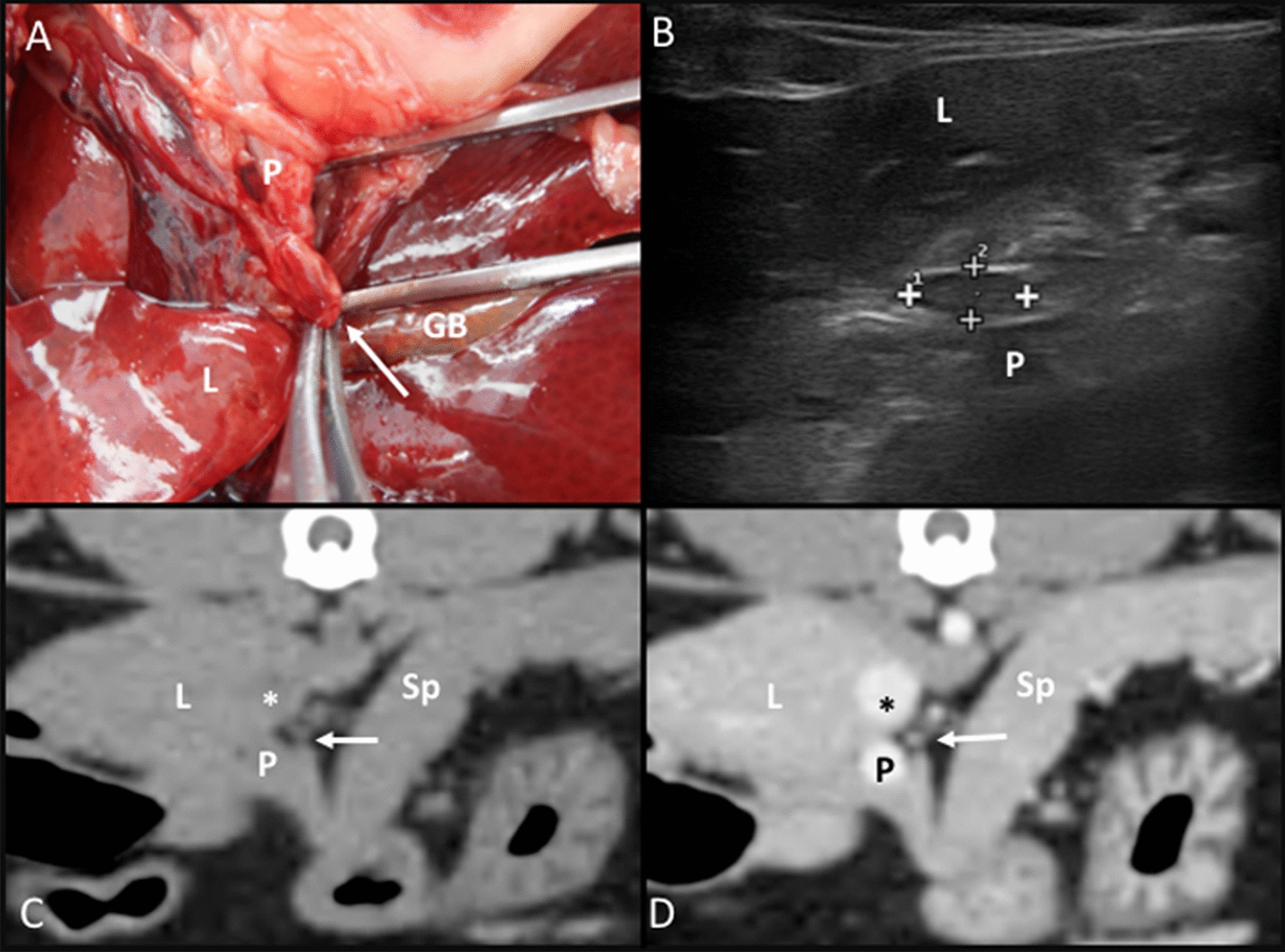
Hepatic lymph node. **A** Image of the dissection showing the localization of the lymph node (arrow) in the *porta hepatis*, near the portal vein (P). The liver (L) and gallbladder (GB) are indicated. **B** Ultrasonographic image showing the hepatic lymph node (between cursors) in the *porta hepatis*. The liver (L) and a partially visible portal vein (P) are indicated. **C**, **D** CT images indicate a slightly hypoattenuating hepatic lymph node (arrow) in the precontrast image (**C**) and with homogeneous contrast enhancement in the postcontrast image (**D**). It is located normally at the dorsomedial aspect of the portal vein (P) and ventromedial to the caudal vena cava (asterisk). The liver (L) and the spleen (Sp) are indicated

Hepatic lymph nodes: In the anatomic study, it was located at the porta hepatis, dorsal, and slightly to the left of the portal vein (Fig. 2A). On the CT transverse images, these lymph nodes were frequently identified dorsally and slightly to the left of the portal vein (Fig. 2B–D). On US images, these were identified by placing the transducer parallel to the spine and caudally to the xiphoid process, fanning slightly from right to left lateral. These lymph nodes were located on the left side of the portal vein. A hyperechoic central line was visible in 6.60% of the lymph nodes.

**Fig. 3:**
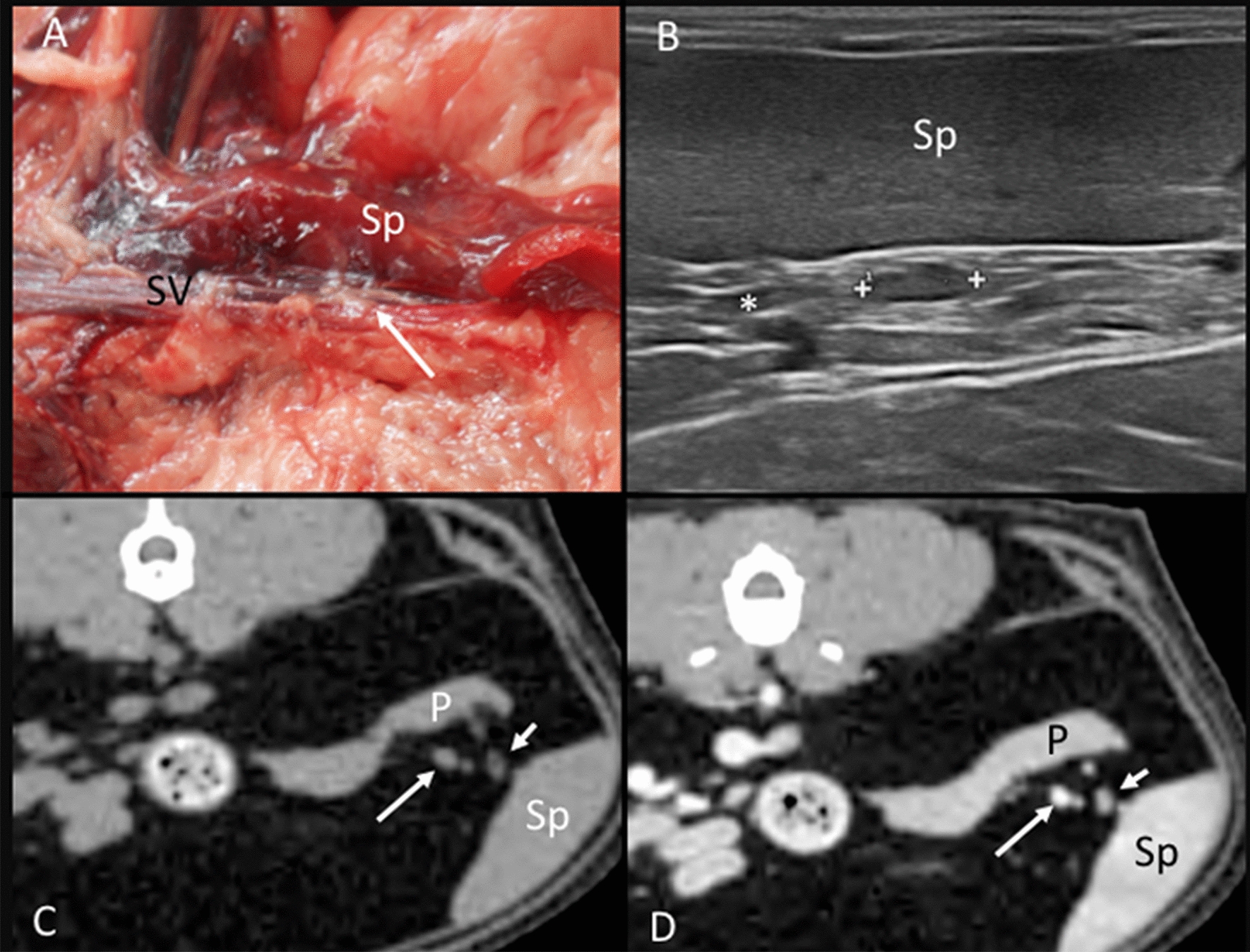
Splenic lymph node. **A** Image of the dissection showing its localization (arrow) in the splenic hilus along the splenic vein (SV). The spleen (Sp) is partially visible. **B** US image showing the splenic lymph node (between cursors) at the splenic hilus. The spleen (Sp) and a partially visible splenic vein (asterisk) are indicated. **C**, **D** CT images indicating the localization of the splenic lymph node (short arrow), which is slightly hypoattenuating with a hypoattenuating center in the precontrast image (**C**) and showing slightly heterogeneous contrast enhancement (**D**). The splenic vein (long arrow), the pancreas (P), and the spleen (Sp) are indicated

Splenic lymph nodes: This lymph node was embedded in the fat tissue of the splenic hilus adjacent to the splenic vein on both the anatomic study and CT images (Fig. 3A, C, D). Ultrasonographically, the splenic vein was the main landmark to localize the lymph node. When identified, this was adjacent to the vein in the fat tissue of the splenic hilus, close to the head of the spleen (Fig. 3B). The appearance of the splenic lymph node was commonly hypoechoic (47.83%). However, a center isoechoic to the mesenteric fat within a hypoechoic periphery was identified in 34.78% of the lymph nodes and was classified as heterogeneous. A hyperechoic central line was identified in 26.70% of the lymph nodes.

**Fig. 4:**
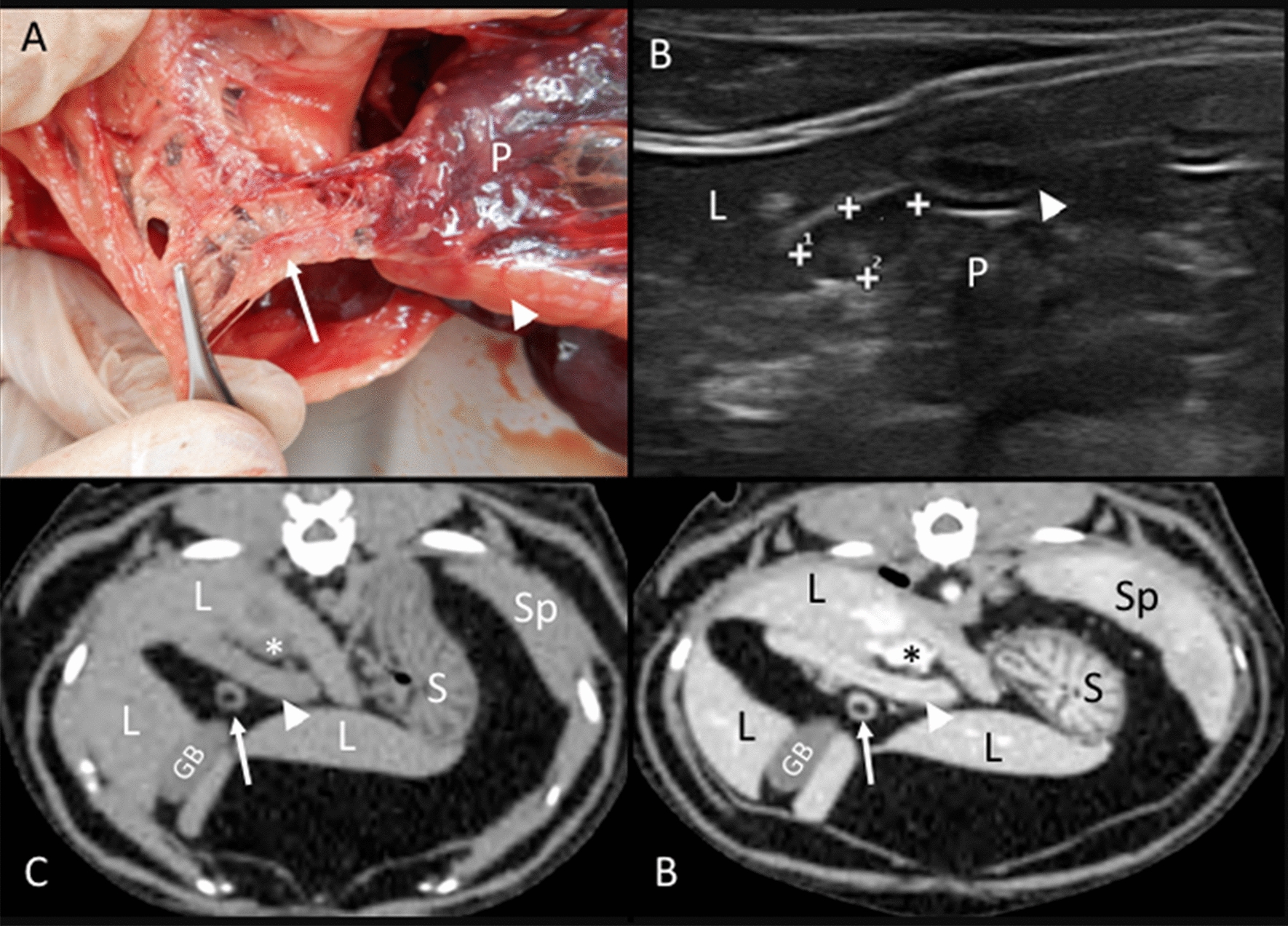
Pancreaticoduodenal lymph node. **A** Image of the dissection showing the localization of the pancreaticoduodenal lymph node (arrow) near the pancreas (P) and the duodenum (arrowhead). **B** US image showing a heterogeneous, rounded pancreaticoduodenal lymph node (between cursors), between the duodenum (arrowhead), pancreas (P), and the liver (L). **C**, **D** CT images indicate the localization of a rounded pancreaticoduodenal lymph node (arrow), which is isoattenuating with a hypoattenuating center in the precontrast image (**C**) and with peripheral homogeneous contrast enhancement in the postcontrast image (**D**). The liver (L), gall bladder (GB), duodenum (arrowhead), portal vein (asterisk), stomach (S), and spleen (Sp) are indicated

Pancreaticoduodenal lymph nodes: In the anatomic study, it was located adjacent to the cranial pancreaticoduodenal vessels at the cranial duodenal flexure (Fig. 4A). On the CT images, one pancreaticoduodenal lymph node was seen ventral to the cranial portion of the duodenum on the right side of the abdomen (Fig. 4C, D). On the US images, one pancreaticoduodenal lymph node was always found on the right side, slightly caudal and ventral to the pylorus and cranial duodenal portion (Fig. 4B). In 37.93% of the identified pancreaticoduodenal lymph nodes, heterogeneous echogenicity was noted, characterized by a central isoechoic to the mesenteric fat area, surrounded by a hypoechoic ring and a thin hyperechoic rim at the periphery.

#### Cranial mesenteric lymph center (*Lymphocentrum mesentericum craniale*)

**Fig. 5:**
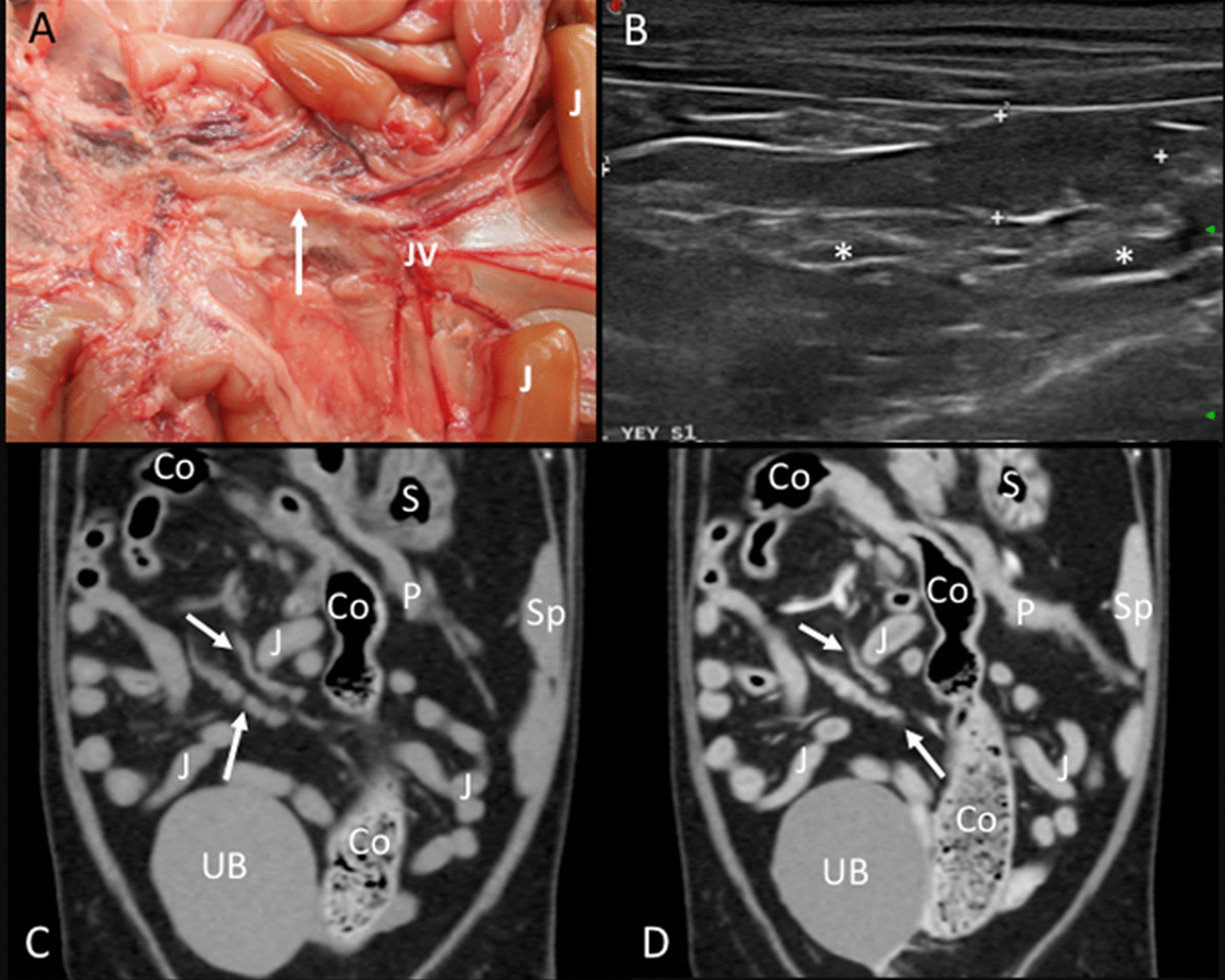
Jejunal lymph nodes. **A** Image of the dissection showing the localization of the jejunal lymph node (arrow) in the mesentery along the jejunal vessels (JV). The jejunal loops (J) are indicated. **B** US image showing a miscellaneous shaped, hypoechoic jejunal lymph node (between cursors). The jejunal vessels (asterisk) are partially seen. **C**, **D** CT images in dorsal reconstruction indicating the localization of the jejunal lymph nodes (arrows) seen isoattenuating in the precontrast image (**C**) and with homogeneous contrast enhancement in the postcontrast image (**D**). The jejunal loops (J), colon (Co), stomach (S), spleen (Sp), pancreas (P), and urinary bladder (UB) are indicated

Jejunal lymph nodes: In the anatomic study, one to four jejunal lymph nodes were identified. Three cadavers presented four lymph nodes, and one, two, or three lymph nodes were found in one cadaver each. These lymph nodes were located along the jejunal vessels just proximal to the origin of the ileocolic artery. At least two large jejunal lymph nodes with an elongated shape were identified in each animal (Fig. 5A). In the CT images, one to three jejunal lymph nodes were frequently (93.3%) identified at the dorsal and ventral aspects of the jejunal vein (Fig. 5C, D). Three jejunal lymph nodes were found in 15 cats, two in 13 cats, and only one lymph node in two cats. On the US examination, the ileocolic junction was used as a landmark. Once it was located, the transducer was directed caudal and to the center of the abdomen until visualizing the jejunal vein. The jejunal lymph nodes were located on each side of the vein. Four jejunal lymph nodes were identified in one cat, three in 16 cats, two in 11 cats, and one lymph node in one cat. A hyperechoic central line was visible in only 5.20% of the jejunal lymph nodes.

**Fig. 6:**
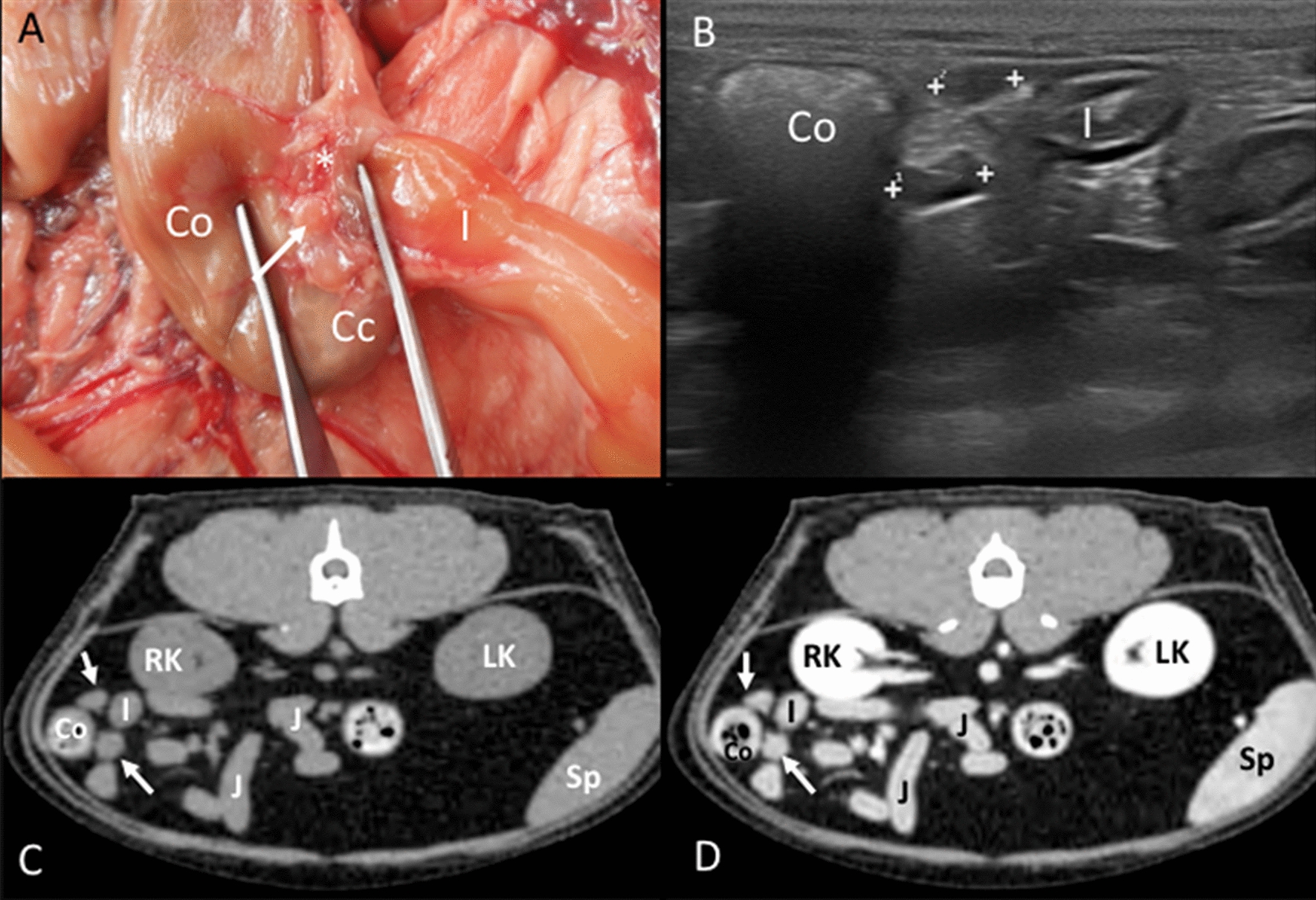
Ileocecal lymph nodes. **A** Image of the dissection showing the localization of the ileocecal lymph nodes (arrow) near the ileocolic junction, along the ileocolic vessels (asterisk). The ileum (I), cecum (Cc), and colon (Co) are indicated. **B** US image showing two elongated and hypoechoic ileocecal lymph nodes (between cursors) between the ascending colon (Co) and ileum (I). **C**, **D** CT images indicating the localization of the ileocecal lymph node (arrows), which is isoattenuating in the precontrast image (**C**) and with homogeneous enhancement in the postcontrast image (**D**). The colon (Co), ileum (I), jejunal loops (J), right (RK) and left (LK) kidneys, and spleen (Sp) are indicated

Ileocecal lymph nodes: In the anatomic study, two lymph nodes were observed in four cadavers, and only one was seen in two cadavers. They were in the ileocecal ligament along the ileocecal vessels, one on each side between the ileum and the cecum (Fig. 6A). On the CT images, two ileocecal lymph nodes were identified in 26 cats and only one in two cats. The anatomic landmark for these lymph nodes was the ileocolic junction. On transverse slices, they were identified slightly caudal to the junction in a dorsal and ventral position between the ileum and cecum (embedded in the adipose tissue of the ileocecal ligament) (Fig. 6C, D). On the US images, two lymph nodes were identified in 24 cats and only one in six cats. The main landmark in finding the ileocecal lymph nodes was the ileocolic junction. An image of this with a sagittal plane of the ileum was obtained, and then a slight movement to the right lateral side of the patient allowed their visualization (Fig. 6B). A hyperechoic central line was visible in 5.50% of the lymph nodes.

**Fig. 7:**
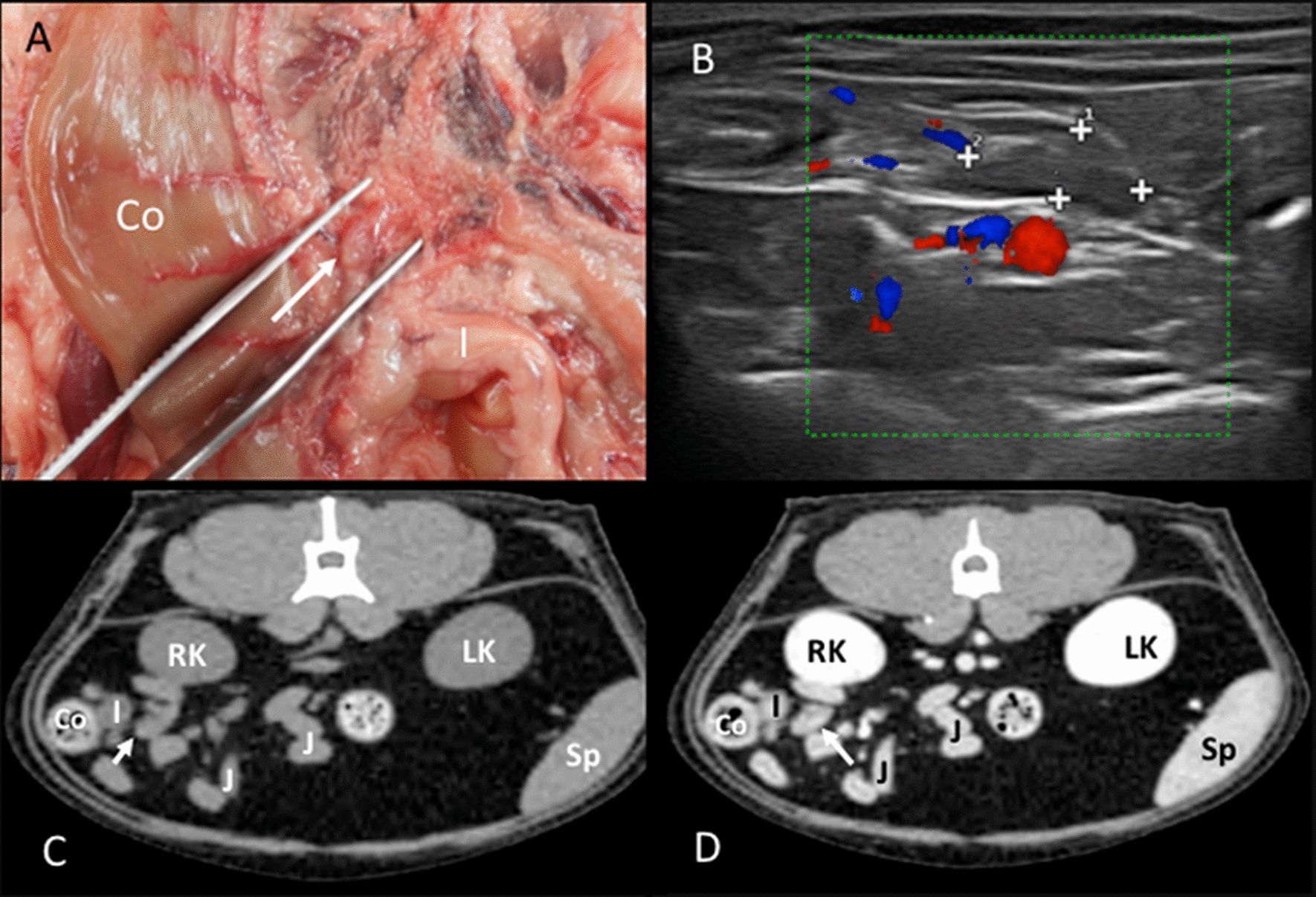
Colic lymph node. **A** Image of the dissection showing the localization of the colic lymph node (arrow) near the ascending colon (Co) and ileum (I). **B** Doppler US image showing the colic lymph node (among cursors), blood flow can be seen in the mesenteric vessels. **C**, **D** CT images indicating the localization of the colic lymph node (arrow), which is isoattenuating in the precontrast image (**C**) and with homogeneous enhancement in the postcontrast image (**D**). The colon (Co), ileum (I), jejunal loops (J), right (RK) and left (LK) kidneys, and spleen (Sp) are indicated

Colic lymph nodes: In the anatomic study, three and five colic lymph nodes were found in two cadavers. Additionally, two and four lymph nodes were seen in one cadaver each. The colic lymph nodes were located in the mesocolon, near the ascending and transverse colon (Fig. 7A). At least two lymph nodes were bigger and more elongated than the rest, normally one near the ascending colon and one within a group of nodes located near the transverse colon. On the CT images, the identification of one, two, three, four, and five colic lymph nodes was possible in twelve, six, four, one, and four cats, respectively. These lymph nodes were seen along the colonic blood vessels (Fig. 7C, D). Multiplanar sagittal reconstructions were helpful in the localization of these lymph nodes. On US images, only one colic lymph node could be successfully identified in 21 cats and was not seen in 9 cats. This lymph node was close to the ileocolic junction (Fig. 7B). In order to distinguish it from the ileocecal lymph nodes, the transducer was displaced medially from the ileocolic junction instead of laterally. A hyperechoic central line was visible in 6.70% of these lymph nodes.

#### Caudal mesenteric lymph center (*Lymphocentrum mesentericum caudale*)

**Fig. 8:**
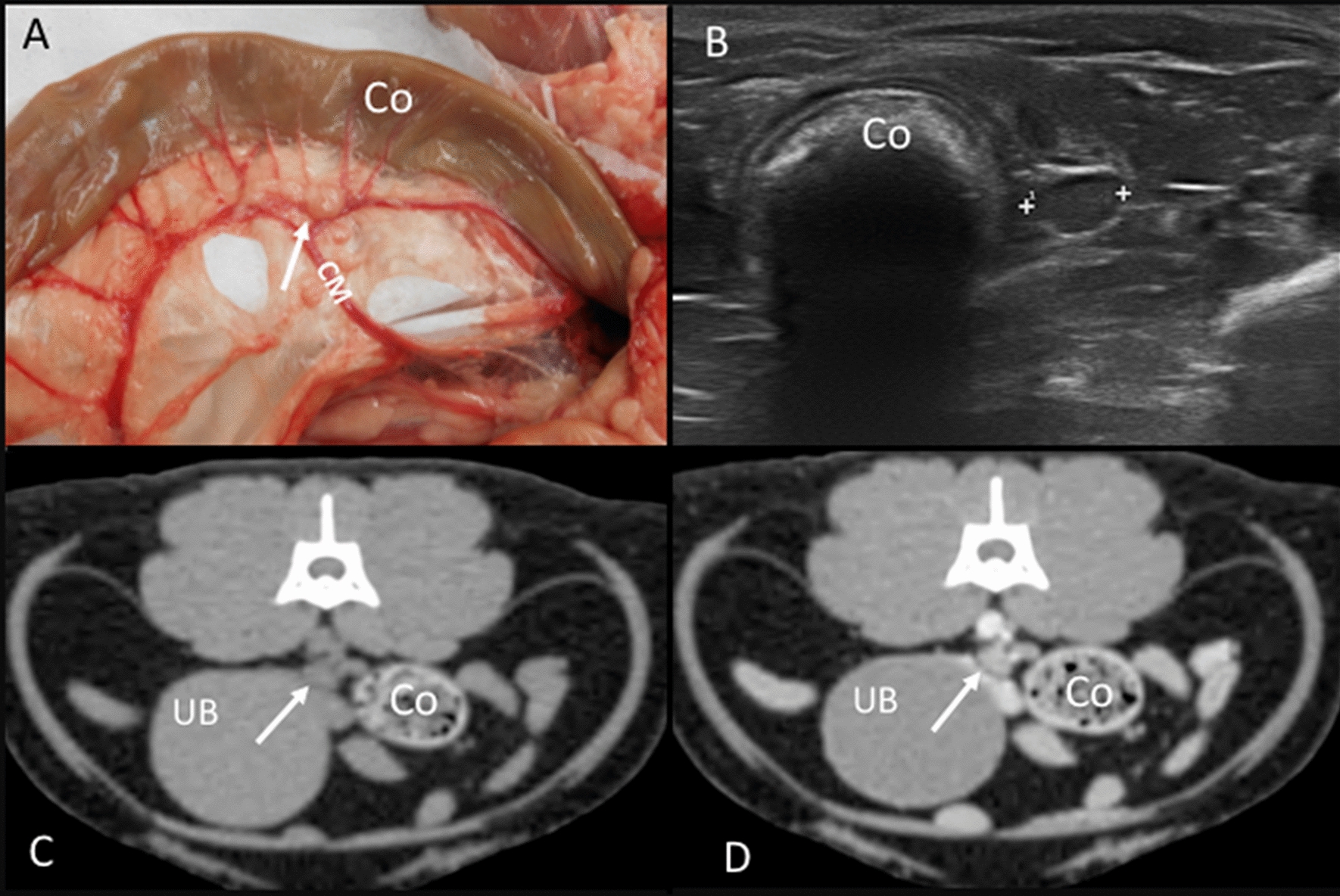
Caudal mesenteric lymph node. **A** Image of the dissection showing the localization of the caudal mesenteric lymph node (arrow) near the descending colon (Co) along the caudal mesenteric vessels (CM). **B** US image showing the caudal mesenteric lymph node (between cursors) near the descending colon (Co). **C**, **D** CT images indicating the localization of the caudal mesenteric lymph node (arrow), which is isoattenuating in the precontrast image (**C**) and with homogeneous enhancement in the postcontrast image (**D**). The descending colon (Co) and the urinary bladder (UB) are indicated

One to four caudal mesenteric lymph nodes were identified in the cadavers of the anatomic study. Two lymph nodes were present in one cadaver. One, three, and four lymph nodes were found in one cadaver each. These lymph nodes were located along the caudal mesenteric vessels (Fig. 8A). On CT images, one lymph node was seen in 14 cats; two, three, and four lymph nodes were found in nine, three, and two cats, respectively. These lymph nodes were seen on transverse images located slightly ventral to the descending colon and dorsolateral to the left aspect of the urinary bladder (Fig. 8C, D). On the US assessment, only one lymph node was identified in 10 of the 30 cats located between the bladder and the descending colon (Fig. 8B). A hyperechoic central line was visible in 3.30% of these lymph nodes.

#### Lumbar lymph center (*Lymphocentrum lumbale*)

**Fig. 9:**
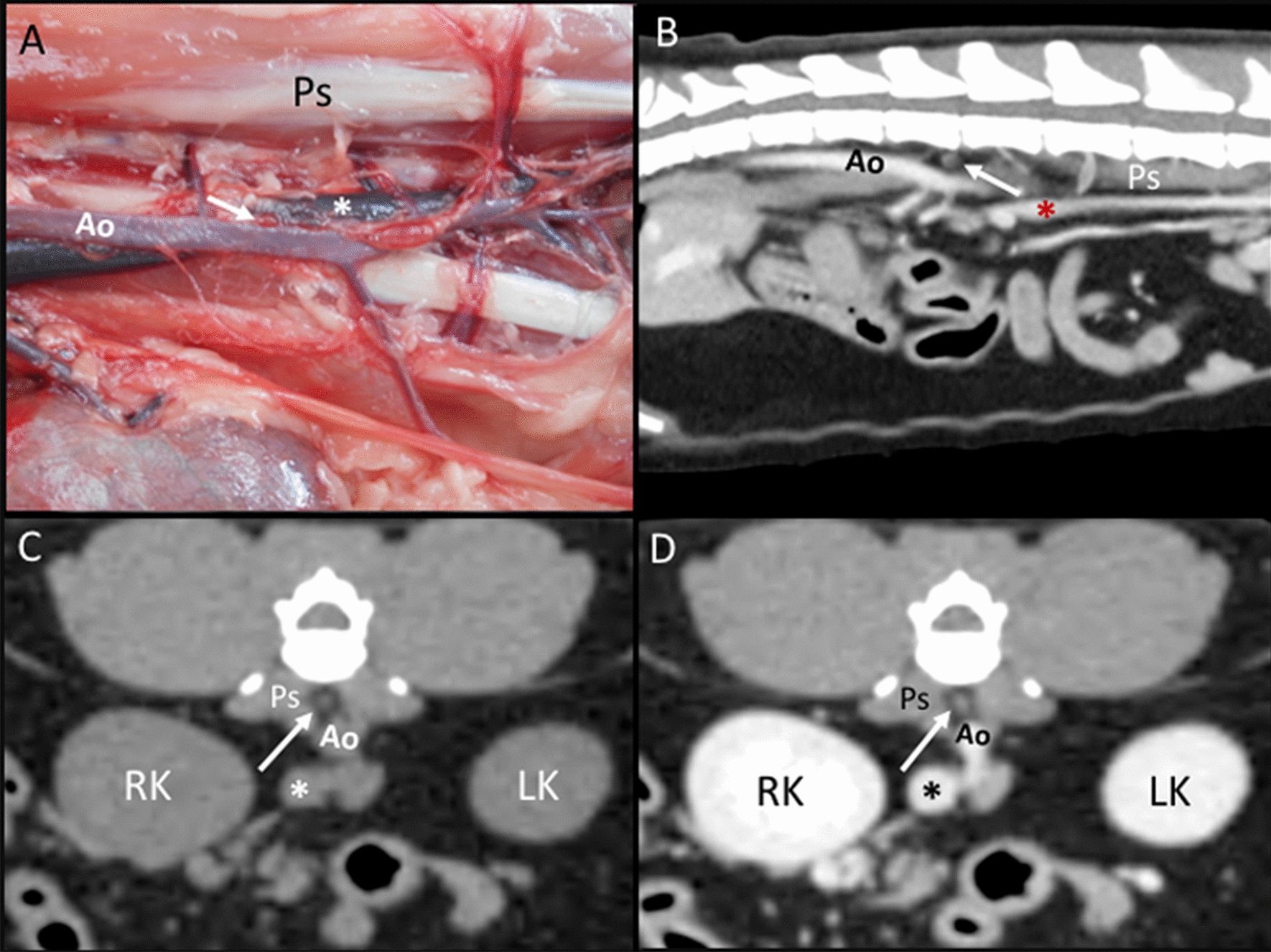
Lumbar aortic lymph nodes. **A** Image of the dissection showing the localization of the lumbar aortic lymph node (arrow) between the abdominal aorta (Ao) and the caudal vena cava (asterisk). The psoas (Ps) muscles are indicated. **B**–**D** CT images indicating the localization of the lumbar aortic lymph node (arrow) in the sagittal plane seen with heterogeneous contrast enhancement with a hypoattenuating central area postcontrast (**B**) and in the transverse plane seen slightly hypoattenuating in the precontrast image (**C**) and with homogeneous enhancement in the postcontrast image (**D**). The psoas (Ps) muscles, aorta (Ao), caudal vena cava (asterisk), and right (RK) and left (LK) kidneys are indicated

Lumbar aortic lymph nodes: In the anatomic study, one, two, and three small rounded lymph nodes were seen in one cadaver each. These lymph nodes were located along and between the aorta and caudal vena cava (Fig. 9A). On CT images, one lumbar aortic lymph node was found in three cats, and two were found in two cats. The presence of fat tissue around the aorta and caudal vena cava in these five cats gave enough contrast to differentiate these lymph nodes from the surrounding soft tissue attenuating structures such as the large vessels or hypaxial musculature. However, the localization of the lumbar aortic lymph nodes on CT images was dorsal to the aorta rather than between the aorta and the caudal vena cava (Fig. 9B–D). The lumbar aortic lymph nodes were not identified ultrasonographically in any of the cats.

Renal lymph nodes: In the anatomic study, the left and right renal lymph nodes could be identified only in two cadavers. These small and rounded lymph nodes were seen in close contact with the renal vessels. On CT images, both renal lymph nodes were identified in five cats, and only the right was seen in two cats. These lymph nodes were located dorsal and slightly cranial to each renal vessel. On the US images, only the right renal lymph node was seen in two cats adjacent to the junction of the renal vein with the caudal vena cava.

#### Iliosacral lymph center (*Lymphocentrum iliosacrale*)

**Fig. 10:**
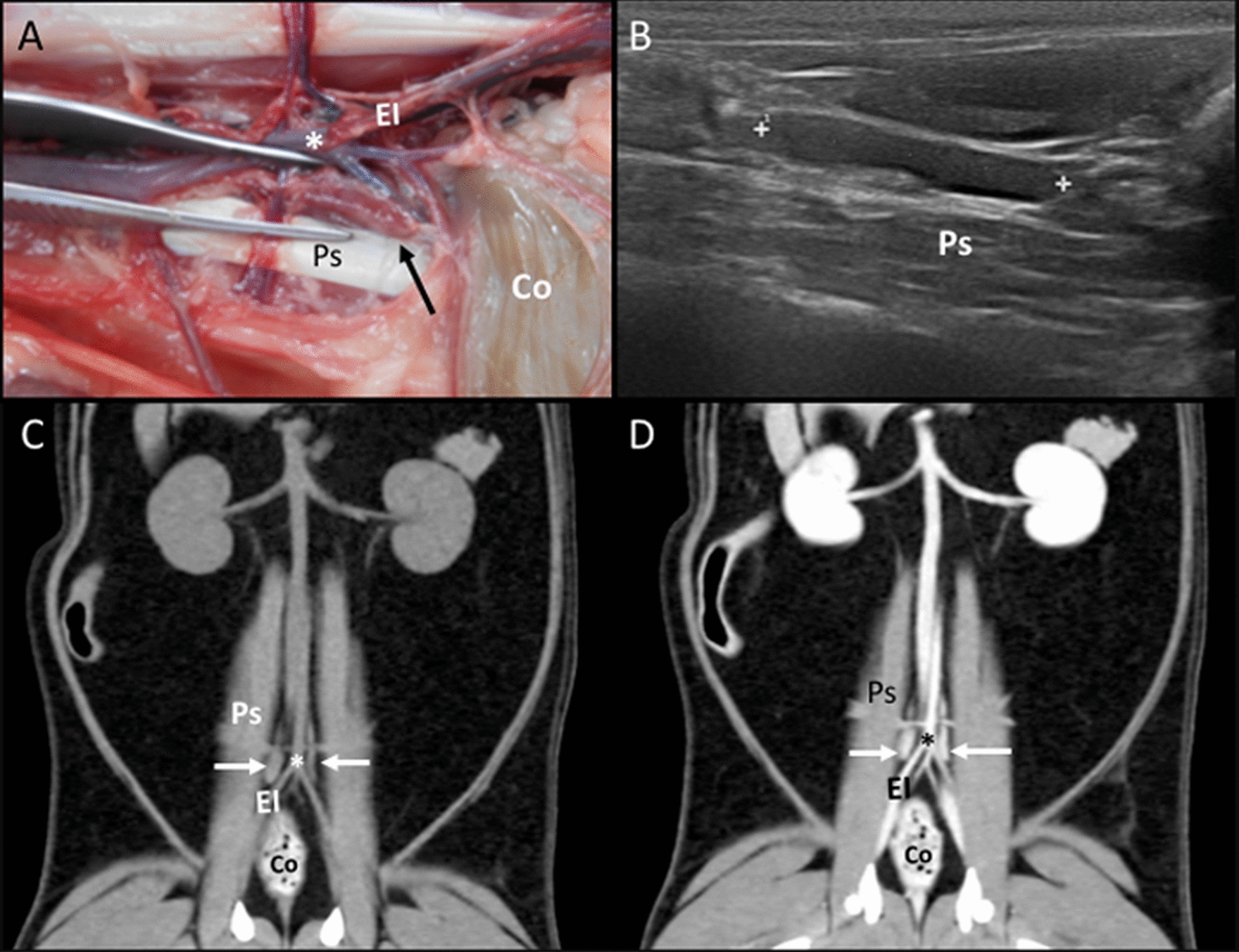
Medial iliac lymph nodes. **A** Image of the dissection showing the localization of the medial iliac lymph node (arrow) along the aortic trifurcation (asterisk) and the external iliac vessels (EI). The psoas muscles (Ps) and descending colon (Co) are indicated. **B** US image showing a hypoechoic medial iliac lymph node (between cursors) with a fusiform shape located ventral to the psoas (Ps) muscles. **C**, **D** CT images indicating the localization of the medial iliac lymph nodes (arrows) in the dorsal plane, which is isoattenuating in the precontrast image (**C**) and with homogeneous enhancement in the postcontrast image (**D**), around the aortic trifurcation (asterisk) between the external iliac vessels (EI) and the psoas (Ps) muscles. The descending colon (Co) is indicated

Medial iliac lymph nodes: In the anatomic study, one right and one left medial iliac lymph node were identified in five cadavers. These singular lymph nodes were located slightly dorsal and lateral to the external iliac arteries and extended cranially until the deep circumflex iliac vessels (Fig. 10A). On the CT images, the lymph nodes were identified in 28 cats bilaterally. The location of these lymph nodes was as described in the anatomic study, and more specifically, extending from the cranial end-plate of the seventh lumbar vertebra (L7) until the cranial end-plate of the first sacral vertebra (S1) (Fig. 10C, D). Some lymph nodes were very thin in their mid-portion, presenting a bilobed shape. Ultrasonographically, both right and left medial iliac lymph nodes were identified in all the cats with a similar location as described in the cadavers and on CT (Fig. 10B). A hyperechoic central line was identified in 18.30% of these lymph nodes.

**Fig. 11:**
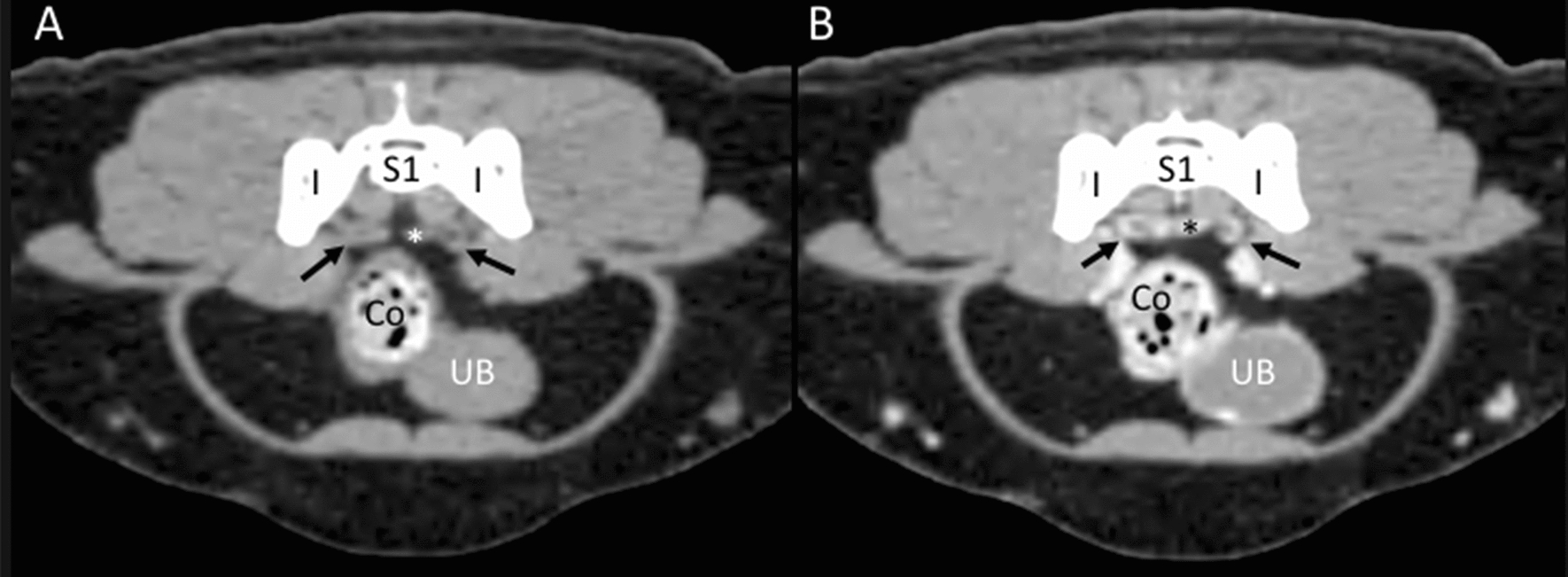
Internal iliac lymph nodes. **A**, **B** CT transverse images indicating the localization of isoattenuating internal iliac lymph nodes (arrows) in the precontrast image (**A**) that show homogeneous enhancement in the postcontrast image (**B**). The lymph nodes are located near the medial aspect of the ilium (I), ventral to the sacrum (S1), and along the internal iliac vessels (asterisk). The colon (Co) and the urinary bladder (UB) are indicated

Internal iliac lymph nodes: Right and left internal iliac lymph nodes were found only in one cadaver, along the internal iliac vessels. On the CT images, these singular lymph nodes were visualized in 17 cats bilaterally. Additionally, unilaterally one right and one left lymph node were identified in two cats each. These lymph nodes were located at the ventrolateral aspect of the first sacral vertebra (ventral to the sacral wings) along the internal iliac vessels in contact with the body of the ilium (Fig. 11). On US assessment, the internal iliac lymph nodes were not identified in any of the cats.

Sacral lymph nodes: These lymph nodes were not identified in the anatomic study. On CT images, the right and the left sacral lymph nodes were identified in two cats. Only the right sacral lymph node was identified in six cats, and only the left was identified in two cats. These lymph nodes were in the midline at the ventral aspect of the sacrum. On US assessment, one right sacral lymph node was visualized in one cat, only in the sagittal plane, allowing the measurement of the length and the height. A transverse image was not possible to obtain due to its caudal position in the pelvic cavity; therefore, the width was not provided.

#### Inguinofemoral lymph center (*Lymphocentrum inguinofemorale*)

**Fig. 12:**
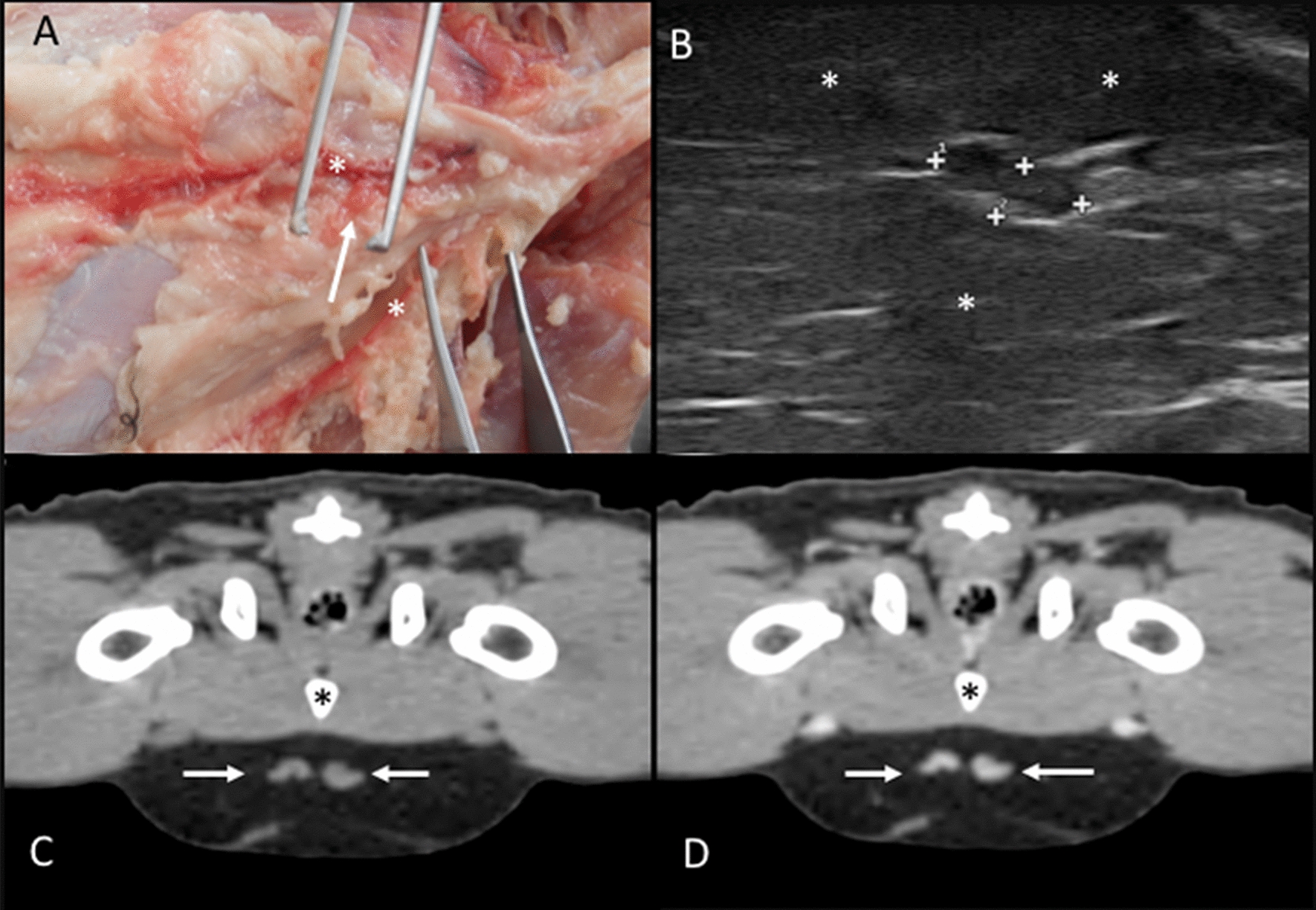
Superficial inguinal lymph nodes. **A** Image of the dissection showing the localization of the superficial inguinal lymph nodes (arrow) along the external pudendal vessels (asterisks). **B** US image showing a heterogeneous superficial inguinal lymph node (between cursors) embedded in the inguinal adipose tissue (asterisks). **C**, **D** CT transverse images indicating the localization of the superficial inguinal lymph nodes (arrows), which is isoattenuating in the precontrast image (**C**) and with homogeneous enhancement in the postcontrast image (**D**). The pubis (asterisk) is indicated

Superficial inguinal lymph nodes: In the anatomic study, the right and the left superficial inguinal lymph nodes were identified in four cadavers. The location of these lymph nodes was cranial to the inguinal canal in contact with the external pudendal vessels and embedded in fat tissue (Fig. 12A). On CT images, the right and left superficial inguinal lymph nodes were identified in 24 cats. Additionally, one right, one left, and one right and two left lymph nodes were seen in one cat each. These lymph nodes were located slightly caudal and dorsal to the junction between the external pudendal and caudal epigastric vessels, ventral to the pubic bone (Fig. 12C, D). On the US assessment, singular right and left superficial inguinal lymph nodes were identified in 28 cats and in one cat only on the left side. They were visualized in the adipose tissue cranioventral to the pubic bone (Fig. 12B). A hyperechoic central line was visible in 21.80% of these lymph nodes.

**Fig. 13:**
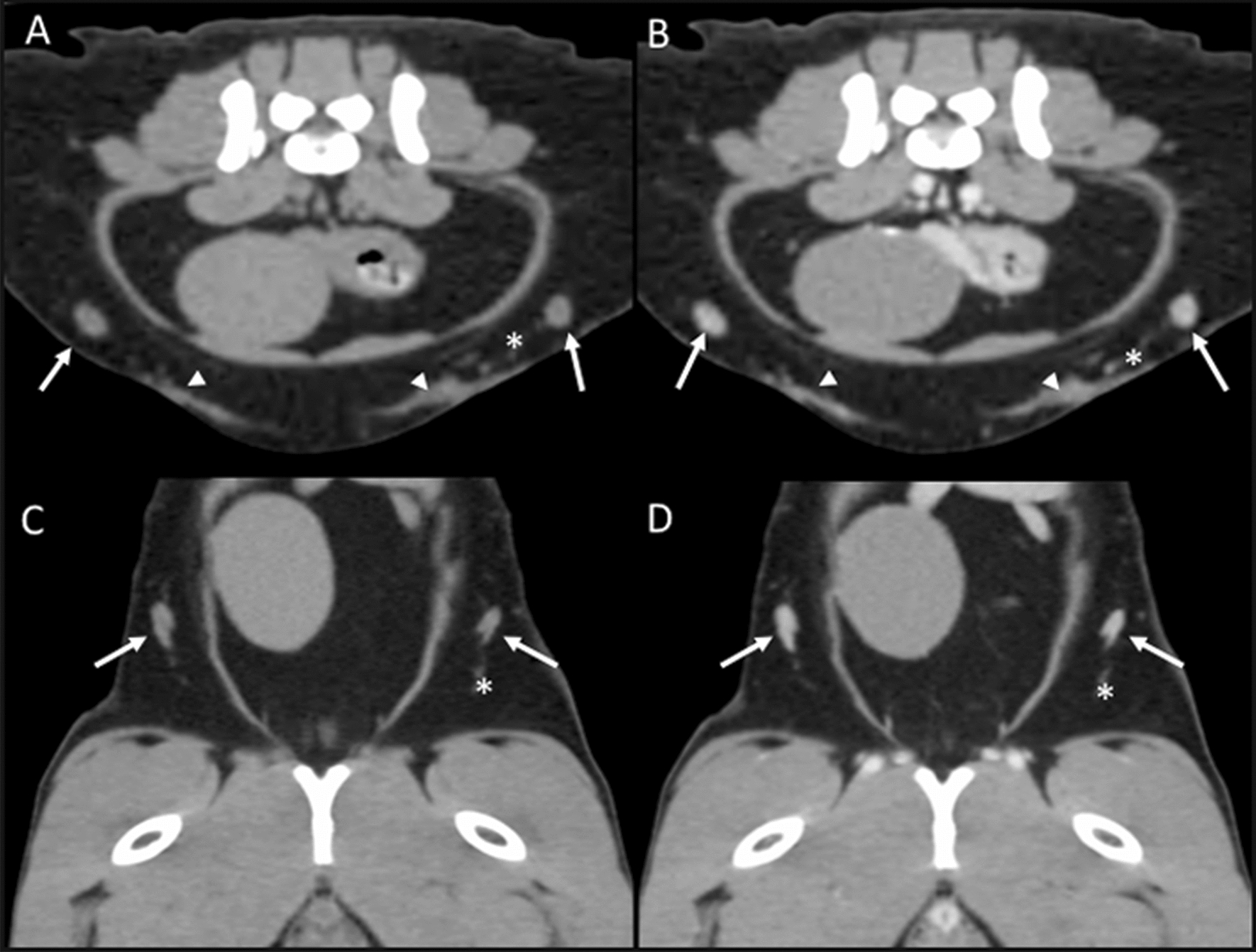
Caudal epigastric lymph nodes. **A**–**D** CT transverse (**A** and **B**) and dorsal (**C** and **D**) images indicating the localization of the caudal epigastric lymph nodes (arrows) in precontrast (**A** and **C**) and postcontrast (**B** and **D**) images along the caudal epigastric vessels (asterisk). In **A** and **B**, mammary tissue is indicated (arrowheads)

Superficial caudal epigastric lymph nodes: This group of lymph nodes was identified in 26 cats only on CT images. One right and occasionally three (in 5/26), and one left and occasionally two (in 2/26) lymph nodes were identified. The location of these lymph nodes was along the caudal epigastric vessels in the subcutaneous adipose tissue of the ventrolateral abdominal wall (at the level of the 6th–7th lumbar vertebrae) (Fig. 13).

#### Ischiatic lymph center (*Lymphocentrum ischiadicum*)

**Fig. 14:**
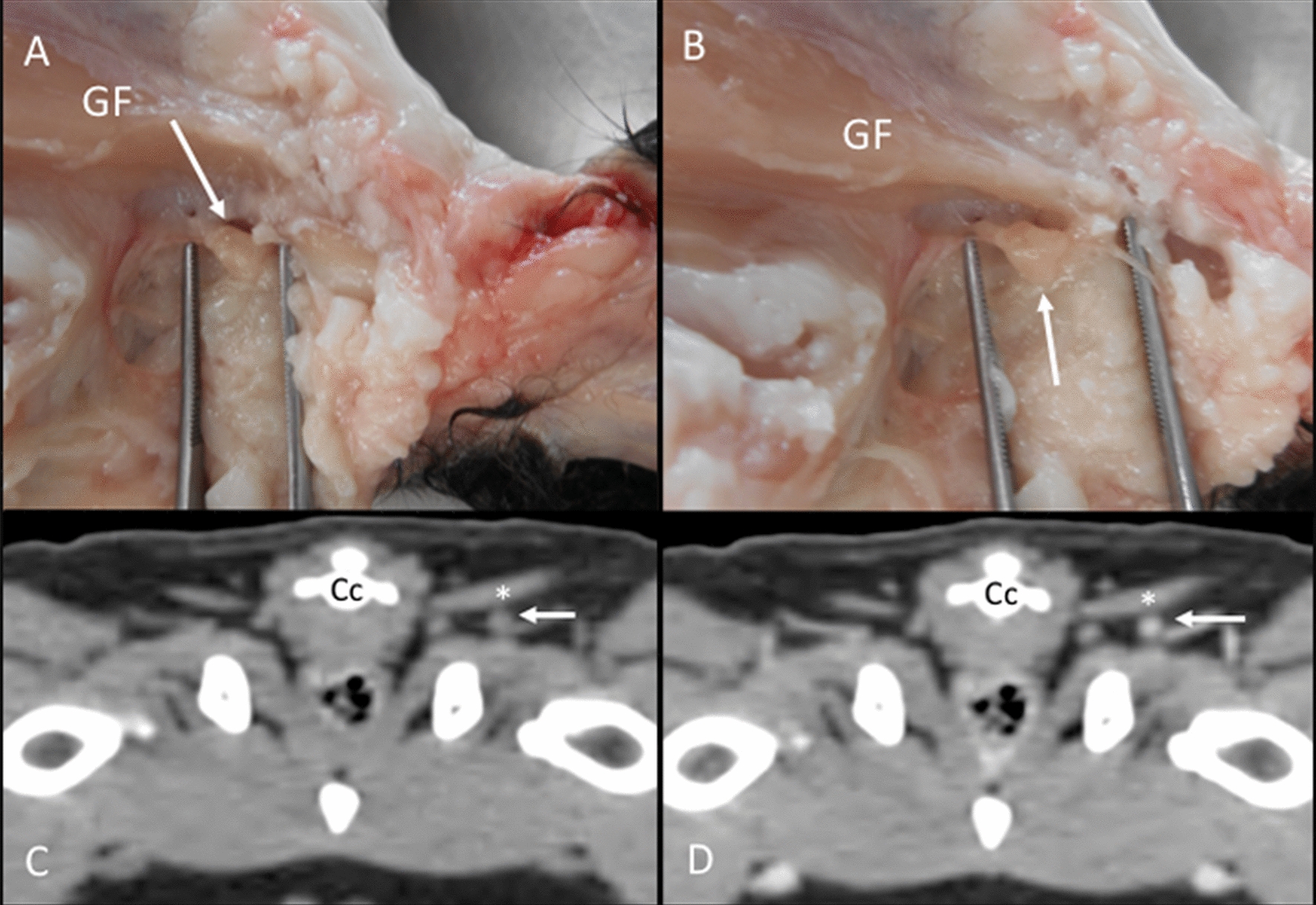
Ischiatic lymph node. **A**, **B** Images of the dissection showing the localization of the ischiatic lymph node (arrow) deep to the gluteofemoralis muscle (GF). **C**, **D** CT transverse images indicating the localization of the ischiatic lymph node (arrow) seen isoattenuating in the precontrast image (**C**) and with homogeneous enhancement in the postcontrast image (**D**), deep to the gluteofemoralis (asterisk) muscle. A coccygeal vertebra (Cc) is indicated

Ischiatic lymph nodes: The right and left ischiatic lymph nodes were found in one cadaver. These rounded nodes were located at the base of the tail, partially covered by the gluteofemoralis muscle and embedded in adipose tissue (Fig. 14A, B). On the CT images, the ischiatic lymph nodes were found in one cat bilaterally. Additionally, five cats presented only the right, and two cats presented only the left lymph node. The location was as described in the anatomic study (Fig. 14C, D). The ischiatic lymph nodes were not visualized with US.

#### Popliteal lymph center (*Lymphocentrum popliteum*)

**Fig. 15:**
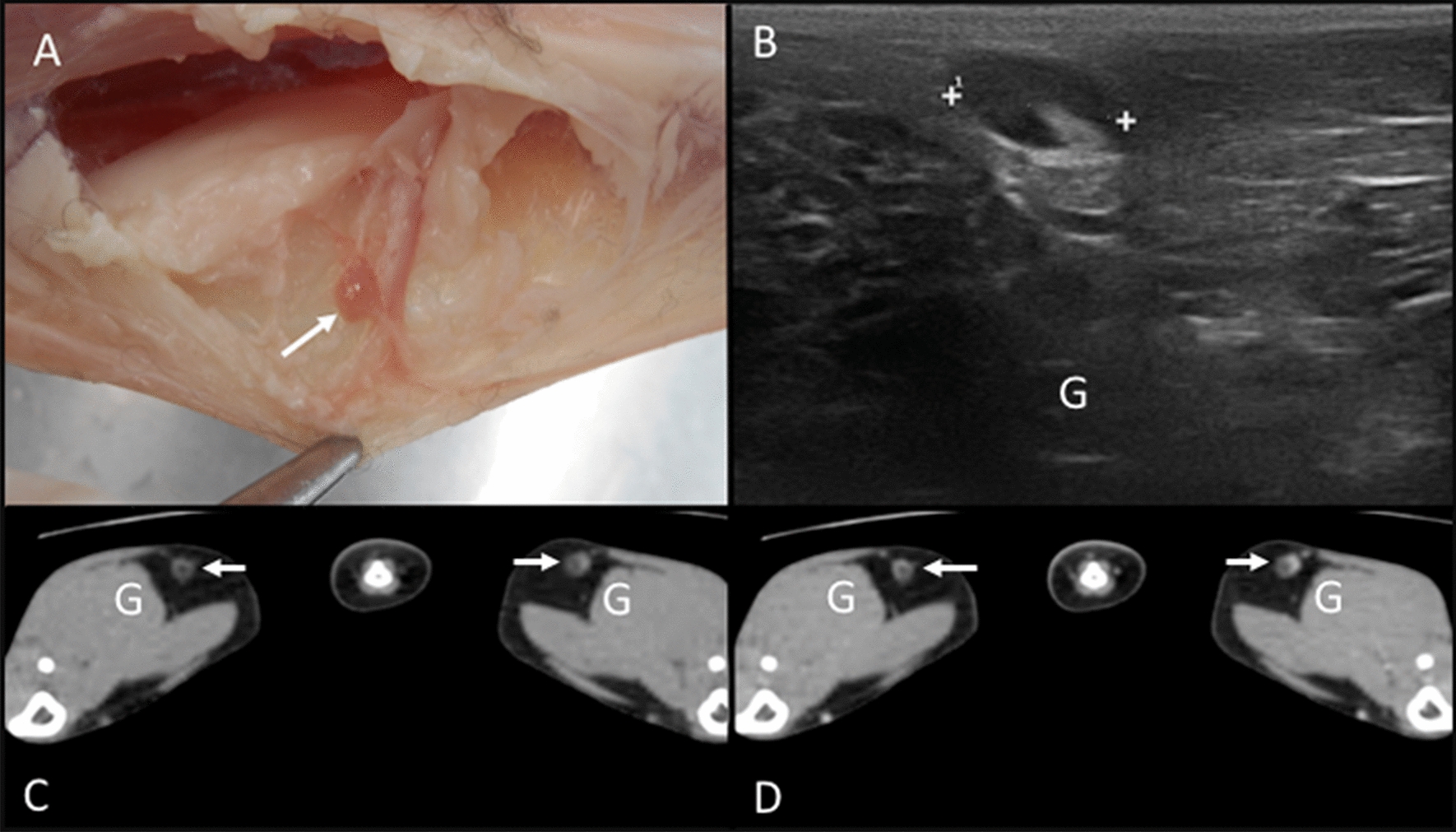
Popliteal lymph nodes. **A**. Image of the dissection showing the localization of the popliteal lymph node (arrow) in the caudo-proximal aspect of the stifle joint. **B**. US image showing a heterogeneous (hyperechoic central area compatible with the hilus) popliteal lymph node (between cursors) caudally to the gastrocnemius muscle (G). **C** - **D**. CT images indicating the localization of the popliteal lymph nodes (arrows) seen as heterogeneous with isoattenuating periphery and hypoattenuating center in the precontrast image (C) with homogeneous peripheral enhancement in the postcontrast image (D). The gastrocnemius (G) muscles are indicated.

The right and left popliteal lymph nodes were identified in all the cadavers. These nodes were embedded in the adipose tissue located in the caudal aspect of the stifle joint, in contact with the medial aspect of the lateral saphenous vein (Fig. 15A). On CT images, both popliteal lymph nodes were identified in 29 cats. On the US assessment, the right and left popliteal lymph nodes were identified in 29 cats, and only the left was seen in one cat. In the imaging study, the popliteal lymph nodes were located in the adipose tissue of the caudolateral aspect of the stifle joints (Fig. 15B–D).

### Statistical analysis

Computed tomography showed a higher frequency of lymph node identification when compared to US and anatomy (Table 1).

The lymph nodes dimensions measured on CT, US, and gross dissection during the anatomic study showed differences when compared to each other using the Wilcoxon signed-rank test (CT and US) and the Mann–Whitney U test (CT, US, and Anatomy) (Tables 2, 3, and 4).

In this study, the MPR-length on CT images showed a better reflection of the dimensions of the abdominal lymph nodes during the anatomy study. Therefore, the MPR-length was chosen to compare between techniques. The comparison of the MPR-length of the lymph nodes showed few differences with US and anatomy mainly with lymph nodes from the celiac and cranial mesenteric lymph center. The length of the hepatic lymph nodes obtained with US was statistically significantly lower when compared to CT and anatomy. Additionally, the length of the jejunal lymph nodes was almost 10 mm higher on CT than on US, and this difference was statistically significant. The length of both ileocecal lymph nodes in anatomy was lower compared to CT and US, showing statistical significance. Although the length of the medial iliac lymph nodes in CT was higher than in anatomy and US, only the length of the lymph node on the right side showed statistically significant differences between CT and anatomy.

The width of the lymph nodes in the anatomic study was often lower than the measurements obtained by CT and US. Statistical significant differences were observed for the pancreaticoduodenal, the second and third jejunal, the ileocecal, the first colic, and the left ischiatic lymph nodes. The width of the gastric lymph node on US was higher compared to CT and anatomy, and the differences were statistically significant. The width of the right medial iliac lymph node in anatomy was slightly higher than on CT, being statistically significant.

The height was the measurement that showed more statistically significant differences among the three techniques. The height of the gastric, the splenic, the pancreaticoduodenal, the jejunal, the left superficial inguinal, and the ileocecal lymph nodes was statistically significantly higher on CT and US than in the cadavers. Besides that, it was statistically different from anatomy height measurements. The height on CT was also statistically significantly higher than on US. Additionally, the height of the hepatic, the colic (except the most caudal), the first caudal mesenteric, and the right medial iliac lymph nodes was statistically significantly higher on CT than with anatomy.

Most of the lymph nodes were isoattenuating or slightly hypoattenuating to the surrounding musculature (Additional file 1). A negative mean Hounsfield unit was obtained in the left renal and lumbar aortic lymph nodes. However, these lymph nodes were tiny, and most of them had a hypoattenuating center and were classified as heterogeneous. After contrast administration, most of the lymph nodes showed homogeneous contrast enhancement. However, the renal lymph nodes (right: n 4/7 = 57.14%; left: n 3/4 = 75.00%) were frequently presenting a heterogeneous attenuation, followed by the splenic (n 5/23 = 21.74%) and both right and left popliteal (n 5/29 = 17.24% each) lymph nodes. A peripheral contrast enhancement was present in eight (n 23) lymph nodes, being more frequent for the splenic lymph nodes.

In ultrasound, the majority of the lymph nodes were hypoechoic, followed by isoechoic. A small percentage showed a center that was isoechoic to the mesenteric fat with a more hypoechoic periphery and a hyperechoic rim; therefore, they were classified as heterogeneous. Mainly the gastric, the splenic, the medial iliac, the superficial inguinal, and the popliteal lymph nodes presented a higher percentage of heterogeneous echogenicity than the other lymph nodes (Additional file 2).

An elongated shape was commonly found in the lymph nodes of this study, however, rounded (hepatic, splenic, pancreaticoduodenal, and popliteal lymph nodes) and miscellaneous (jejunal, medial iliac, and ileocecal lymph nodes) shapes were also present (Additional file 3).

## Discussion

The identification of almost all the lymph centers of the abdomen, pelvis, and hindlimb was possible during gross dissection in the anatomic study. However, accurate assessment of some lymph nodes (e.g., lumbar aortic, sacral, and caudal epigastric lymph nodes) was not achieved because of their similar appearance to the surrounding fat tissue. A dyeing procedure was not performed before the dissection because the animals were presented for post-mortem examination within 24 h of death. In our study, the mean length of the abdominal lymph nodes in the anatomic study was smaller than the reports in the literature. Previous anatomic descriptions reported a length range for the abdominal lymph nodes of 1 cm to 8 cm. However, that study included cats from 7 days to 10 years old, resulting in 33.3% of cats under 1 year old [16]. Previous studies reported statistically significant differences in the size of lymph nodes when comparing young versus adult animals because of the active production of lymphoid cells at a young age [13, 14].

The lymph nodes of the abdomen, pelvis, and hindlimbs in dogs have been well documented in the anatomy books and assessed using CT and US [1, 2, 4, 10, 15]. In cats, the anatomic locations, landmarks, drainage areas, and the number of lymph nodes per lymph center have been well documented in anatomy studies [3, 4, 16, 12]. However, in those anatomic references, mostly the length of lymph nodes was reported. In our study, we report not only the length of the abdominal and hindlimb lymph nodes but their width and height as well.

A previous study reported the CT features of the abdominal lymph nodes in healthy cats [17]. However, the paper refers to each lymph node in the abdomen as the lymph centers. In our study, a clear definition of each lymph center and its representative lymph nodes is made. Also, in the mentioned study, the dimensions and features from several lymph nodes per location (e.g., three or more jejunal lymph nodes) were combined to provide the mean values. In our study, a description of the number of lymph nodes per location was provided. This may explain the difference in length and width (smaller values in the previous study) when compared to the lymph nodes in our study. Additionally, it only reports the length and width, but there is no clarity on how these measurements were obtained or the landmarks used to differentiate each group of lymph nodes.

A description of the ultrasonographic characteristics of the lymph nodes in the abdominal cavity was previously published [5]. In that study, the visualization of the medial iliac, jejunal, pancreaticoduodenal, splenic, and lumbar aortic lymph nodes in the cat was reported with a frequency between 60 and 100% [5]. In contrast, the ileocecal, colic, and gastric lymph nodes were also identified with a high frequency in this study. Additionally, the caudal mesenteric lymph node was also more frequently identified in our study than in the previously published study [5]. The lumbar aortic, internal iliac, left sacral, and left renal lymph nodes were not visualized on US images in our study, but they were identified in a previous study [5]. We hypothesize that this could be due to the lack of contrast between these small structures and the tissues between the aorta and caudal vena cava. The use of real-time compound imaging US in the previous publication, which produces a higher border definition and soft tissue contrast compared with B-Mode, might have increased their ability to depict these lymph nodes [5].

The popliteal lymph node was assessed in one study during ultrasound-guided intranodal injection of contrast medium as part of a thoracic duct lymphography in cats using CT [8]. The US features were not provided, but their measurements were similar to those in the present study.

Few statistical differences were found regarding the length of the lymph nodes among techniques. The hepatic and jejunal lymph nodes were shorter on US than in the other techniques. We hypothesized that the presence of gas in the stomach and intestinal loops, the location (hepatic lymph node), and a miscellaneous shape (jejunal lymph nodes) could have influenced the achievement of a correct acoustic window to obtain the whole length of these lymph nodes. Similar limitations have been reported in dogs [18]. The mean length of the lymph nodes on US in our study showed some differences from those previously reported [5]. The hepatic and caudal mesenteric lymph nodes in our study were longer. Additionally, the pancreaticoduodenal and right sacral lymph nodes in our study were shorter. The cause for these differences remains unclear, but the sample size, demographic characteristics of included cats, fasting period, the scanning planes, and the interobserver variability might have been contributing factors.

The width and height of the lymph nodes obtained in the anatomic study were shorter than in the imaging techniques. Some of them showed statistical differences, mainly for the pancreaticoduodenal, jejunal, ileocecal, and colic lymph nodes. We hypothesized that the lack of blood perfusion and the loss of fluids after death could contribute to these differences.

The height was the most variable measurement between CT and US. A possible explanation is that the relative orientation of the lymph nodes is likely to be influenced by patient position, scanning plane used for the assessment, and filled intestinal loops and peristalsis that could induce displacement in a ventrodorsal or lateral direction of the lymph nodes. All the cats in this study were positioned in dorsal recumbency for the acquisition of the CT images. However, avoiding an oblique orientation of the abdominal lymph nodes in CT transverse images was challenging. Similar limitations have been described in dogs [2].

The attenuation and Hounsfield Units of the lymph nodes in this study before contrast administration was consistent with the descriptions and values reported for dogs [2] and the medial retropharyngeal lymph nodes in healthy cats [9]. In our study, some of the lymph nodes in the abdominal cavity (splenic and renal) and in the hindlimb (popliteal) showed a nodal periphery isoattenuating or slightly hypoattenuating to the musculature; meanwhile, the center of the node was hypoattenuating. This appearance has been reported for abdominal lymph nodes in dogs [2, 15, 19], and has been observed by these authors for the sternal and axillary lymph nodes in healthy cats, and is produced by the presence of a fatty hilus [11]. After contrast administration, a homogeneous contrast enhancement was more frequently visualized. In the lymph nodes with a fatty hilus, a peripheral enhancement was observed. This phenomenon is due to the presence of vascularized nodal tissue in the periphery; meanwhile, the hilus had less vascularized fat tissue.

The lymph nodes of the abdomen, pelvis, and hindlimb were more frequently hypoechoic or isoechoic to the surrounding tissue. Similar descriptions have been reported for dogs [18]. The presence of a hyperechoic center, especially in the popliteal lymph nodes, was most likely due to fat in the hilus (a feature also visible in CT). This hyperechoic center was different from the described hyperechoic central line. This line was thin and well-defined, corresponding to the description of the hilus [6, 7, 9, 20].

The shape of the lymph nodes in this study is similar to previously reported shapes for dogs [6, 7, 14, 18, 21]. The lymph nodes that presented a rounded shape were smaller and had regular margins. The shape could be compared with the image in CT, being the same in all the cases. In previous reports, rounded lymph nodes in combination with increased size, loss of the hilus, and echotextural changes were suggestive of malignancy [10, 22, 23]. The rounded lymph nodes in our study were small, regular, and homogeneous and were considered normal since all the included cats were healthy according to physical examination and blood tests.

The present study included several limitations. The search for feline cadavers with a cause of death other than neoplastic or inflammatory diseases was challenging, resulting in a small sample size in the anatomic study. The use of ink solutions or other staining procedures in the cadavers was not performed, which could have assisted in the differentiation of the lymph nodes from the surrounding fat tissue during dissection, especially in the sublumbar region and for the superficial inguinal lymph nodes. The animals used in the imaging study were assessed with US immediately after the CT scan. Therefore, the analysis of the CT images was not performed at the same time as the US examination, making an exact correlation in the number of lymph nodes identified with both techniques challenging. In some of the patients, a period of apnea during the whole-body CT scan was difficult to achieve, and movement artifact was present, especially in the cranial abdomen. This artifact reduced the identification of the lymph nodes, mainly from the celiac lymph center. All the cats included in this study were fasted to avoid complications during anesthesia; however, some of them presented gas and feces in the colon that may have reduced the visualization of the lymph nodes of the lumbar and iliosacral lymph centers on US due to the gas and fecal material reverberation and distal acoustic shadowing artifacts.

## Conclusions

The identification of the lymph nodes of the abdomen, pelvis, and hindlimb is excellent with both imaging techniques when compared with node identification during gross dissection. The length of the lymph nodes is more challenging to assess with US than with CT, where multiplanar reconstruction can be used. The lymph nodes were more frequently isoattenuating to surrounding musculature in CT. However, some of them showed a hypoattenuating center corresponding to a fatty hilus. Frequently, the abdominal and hindlimb lymph nodes were hypoechoic or isoechoic. An elongated shape with regular margins was most frequently visible except for the jejunal, medial iliac, and ileocecal lymph nodes that showed a miscellaneous shape and the hepatic, splenic, pancreaticoduodenal, and popliteal lymph nodes that were rounded. This study can be used as an anatomic and imaging reference while comparing the size of abdominal lymph nodes in cadavers, US, and CT examinations, as well as provide a detailed description of the lymph centers in CT and US.

## Supplementary Information

**Additional file 1.** Computed tomography characteristics of the lymph nodes of the abdomen, pelvis, and hindlimb of healthy cats.

**Additional file 2.** Ultrasonographic features of the lymph nodes of the abdomen, pelvis, and hindlimb in healthy cats.

**Additional file 3.** Distribution of the different shapes of the lymph nodes in each technique according to the short-to-length axis ratio.

## Data Availability

The datasets obtained and analyzed during the current study are available from the corresponding author on reasonable request.

## References

[1] Bezuidenhout AJ, Evans HE, De Lahunta A (2013). The lymphatic system. Miller’s anatomy of the dog.

[2] Beukers M, Vilaplana Grosso F, Voorhout G (2013). Computed tomographic characteristics of presumed normal canine abdominal lymph nodes. Vet Radiol Ultrasound.

[3] Saar LI, Getty R, Getty R (1982). Sistema linfático de los carnívoros. Sisson S–Grossman JD Anatomía de los animales domesticos.

[4] International committee veterinary gross anatomical nomenclature. Nomina anatómica veterinaria. 2017;160.

[5] Schreurs E, Vermote K, Barberet V, Daminet S, Rudorf H, Saunders JH (2008). Ultrasonographic anatomy of abdominal lymph nodes in the normal cat. Vet Radiol Ultrasound.

[6] D’Anjou M-A, Penninck D, D’Anjou M-A (2015). Abdominal cavity, lymph nodes, and great vessels. Atlas of small animal ultrasonography.

[7] Nyman HT, O’Brien RT (2007). The sonographic evaluation of lymph nodes. Clin Tech Small Anim Pract.

[8] Lee N, Won S, Choi MM, Kim J, Yi K, Chang D (2012). CT thoracic duct lymphography in cats by popliteal lymph node iohexol injection. Vet Radiol Ultrasound.

[9] Nemanic S, Nelson NC (2012). Ultrasonography and noncontrast computed tomography of medial retropharyngeal lymph nodes in healthy cats. Am J Vet Res.

[10] Nyman HT, Kristensen AT, Skovgaard IM, McEvoy FJ (2005). Characterization of normal and abnormal canine superficial lymph nodes using gray-scale B-mode, color flow mapping, power, and spectral doppler ultrasonography: A multivariate study. Vet Radiol Ultrasound.

[11] Tobón Restrepo M, Espada Y, Aguilar A, Moll X, Novellas R (2021). Anatomic, computed tomographic, and ultrasonographic assessment of the lymph nodes in presumed healthy adult cats: the head, neck, thorax, and forelimb. J Anat.

[12] Sugimura M, Kudo N, Takahata K. Studies on the lymphonodi of cats: III. Macroscopical observations on the lymphonodi in the abdominal and pelvic cavities. Jpn J Vet Res. 1958;6:69–88. http://hdl.handle.net/2115/1729. Accessed 1 Oct 2013.

[13] Burns GO, Scrivani PV, Thompson MS, Erb HN (2008). Relation between age, body weight, and medial retropharyngeal lymph node size in apparently healthy dogs. Vet Radiol Ultrasound.

[14] Krol L, O’Brien R (2012). Ultrasonographic assessment of abdominal lymph nodes in puppies. Vet Radiol Ultrasound.

[15] Rossi F, Patsikas MN, Wisner ER, Schwarz T, Saunders JH (2011). Abdominal lymph nodes and lymphatic collecting system. Veterinary computed tomography.

[16] Sugimura M, Kudo N, Takahata K. Studies of lymphonodi of cats: II. Macroscopical observations on the lymphonodi of the body surfaces, thoracic and pelvic limbs. Jpn J Vet Res. 1956;4:101–12. http://hdl.handle.net/2115/1694. Accessed 1 Oct 2013.

[17] Perlini M, Bugbee A, Secrest S, Scott SC (2018). Computed tomographic appearance of abdominal lymph nodes in healthy cats. J Vet Intern Med.

[18] Agthe P, Caine AR, Posch B, Herrtage ME (2009). Ultrasonographic appearance of jejunal lymph nodes in dogs without clinical signs of gastrointestinal disease. Vet Radiol Ultrasound.

[19] Kneissl S, Probst A (2007). Comparison of computed tomographic images of normal cranial and upper cervical lymph nodes with corresponding E12 plastinated-embedded sections in the dog. Vet J.

[20] Tobón Restrepo M, Novellas Torroja R, Dominguez Miño E, Espada Gerlach Y, Martínez Pereira Y, Tobón Restrepo M (2015). Ultrasound of the abdominal cavity, lymph nodes and large vessels. Diagnostic ultrasound in cats.

[21] Llabres-Diaz FJ (2004). Ultrasonography of the medial iliac lymph nodes in the dog. Vet Radiol Ultrasound.

[22] De Swarte M, Alexander K, Rannou B, D’Anjou M-AA, Blond L, Beauchamp G (2011). Comparison of sonographic features of benign and neoplastic deep lymph nodes in dogs. Vet Radiol Ultrasound.

[23] Dennis R, Kirberger RM, Barr F, Wrigley RH (2010). Handbook of small animal radiology and ultrasound: techniques and differential diagnoses.

